# High-capacity embedding of synfire chains in a cortical network model

**DOI:** 10.1007/s10827-012-0413-9

**Published:** 2012-08-11

**Authors:** Chris Trengove, Cees van Leeuwen, Markus Diesmann

**Affiliations:** 1 Integrated Simulation of Living Matter Group, RIKEN, Computational Science Research Program, Wako, Saitama Japan; 2 Laboratory for Perceptual Dynamics, RIKEN, Brain Science Institute, Wako, Saitama Japan; 3 Laboratory for Computational Neurophysics, RIKEN, Brain Science Institute, Wako, Saitama Japan; 4 Perceptual Dynamics Laboratory, University of Leuven, Tiensestraat 102, 3000, Leuven, Belgium; 5 Institute of Neuroscience and Medicine, Computational and Systems Neuroscience (INM-6), Research Center Juelich, Juelich, Germany

**Keywords:** Recurrent network dynamics, Feedforward network, Synchrony, Synaptic conductance, Synfire chain, Storage capacity

## Abstract

Synfire chains, sequences of pools linked by feedforward connections, support the propagation of precisely timed spike sequences, or synfire waves. An important question remains, how synfire chains can efficiently be embedded in cortical architecture. We present a model of synfire chain embedding in a cortical scale recurrent network using conductance-based synapses, balanced chains, and variable transmission delays. The network attains substantially higher embedding capacities than previous spiking neuron models and allows all its connections to be used for embedding. The number of waves in the model is regulated by recurrent background noise. We computationally explore the embedding capacity limit, and use a mean field analysis to describe the equilibrium state. Simulations confirm the mean field analysis over broad ranges of pool sizes and connectivity levels; the number of pools embedded in the system trades off against the firing rate and the number of waves. An optimal inhibition level balances the conflicting requirements of stable synfire propagation and limited response to background noise. A simplified analysis shows that the present conductance-based synapses achieve higher contrast between the responses to synfire input and background noise compared to current-based synapses, while regulation of wave numbers is traced to the use of variable transmission delays.

## Introduction

Evidence of precisely-timed spiking activity suggests that *synfire chains* play an important role in representing information in the brain (Abeles 1982; Riehle et al. 1997; Prut et al. 1998; Shmiel et al. 2005; Kilavik et al. 2009; Long et al. 2010). Synfire chains, pools of neurons sequentially linked by feed-forward connections, are capable of generating temporally extended and precisely timed patterns of spiking activity. Each such pattern consists of a packet of near-simultaneous spikes within a pool, that triggers a similar packet in the subsequent pool, and so on, giving rise to wave-like propagation of spiking activity down the chain (Abeles 1982). Propagation of synfire waves is robust and efficient because of the properties of single model neurons: a relatively small number of simultaneously arriving inputs can elicit a spike reliably with precise timing (Abeles 1991; Diesmann et al. 1999; Goedeke and Diesmann 2008).

Several authors have presented models of synfire chains as embedded in the detailed structure of the cortex i.e. its synaptic connections and strengths (Bienenstock 1995; Herrmann et al. 1995; Mehring et al. 2003; Aviel et al. 2005; Kumar et al. 2008; Schrader et al. 2008). Modeling of embedding involves assigning individual neurons randomly to pools which are then linked up to form one or more synfire chains. Individual units can belong to more than one pool and thus participate in several different chains, thereby forming a recurrent network.

A system of many embedded synfire chains may provide a form of sparse coding (Földiák 2002), where each chain represents some feature of a cognitive representation. By enabling activation of chains in parallel or in cascaded fashion, embedding can in principle realize combinatorial representation (Bienenstock 1991, 1995; Bienenstock 1996; Abeles et al. 2004; Hayon et al. 2005; Hanuschkin et al. 2010; Schrader et al. 2010).

To permit combinatorial representations, a system needs to, first, embed a sufficient number of pools and, second, allow a sufficient number of these to be activated simultaneously. Let us consider the number of chains or, more specifically, the number of pools they consist of. The number of pools is constrained by factors such as the number of synapses, and the need to avoid excessive overlap between pools. The severity of these constraints is determined by the dynamics of neural activity; the dynamics must enable selective initiation and stable propagation of synfire waves, and avoid modes that would interfere with wave propagation, such as ones characterized by excessively high activity levels or strong oscillations. These dynamical constraints put a limit on how many pools, or more precisely, how many pool-to-pool links can be embedded in the system. This we call the system’s embedding capacity.

A model of cortical embedding of chains should operate at a biologically realistic level of neural connectivity: of the order of 10^4^ synapses per neuron. An early model of chain embedding in cortex achieved an embedding capacity of about 8*N* pools in a network of *N* neurons with cortical scale connectivity (Bienenstock 1995). However, the model presented by Bienenstock is rather abstract: it consists of binary state neurons with an *r*-winners-take-all update scheme that enforces a low level of activity in the network (exactly *r*/*N* at each time-step), and a coarse discretization of time that artificially enforces spike packet synchronization. A similarly idealized model using a global inhibitory signal instead of *r*-winners-take-all gives quantitatively similar embedding capacity results (Herrmann et al. 1995).

Independently, studies using leaky integrate-and-fire neurons with fine temporal resolution have shown that spike packet sequences can propagate while conserving both the number of spikes and narrow temporal dispersion within each packet (Diesmann et al. 1999; Goedeke and Diesmann 2008). However, the high embedding capacity that is found in the idealized models of Bienenstock and Herrmann et al. has proved difficult to realize using integrate-and-fire neurons, due to the appearance of global modes of activity incompatible with wave propagation. The state of the art thus leaves room for improvement. Our first aim is, therefore, to show that such realization is possible.

Like all these models of chain embedding, ours takes as its starting point the balanced random network architecture. This architecture consists of two populations of neurons, inhibitory and excitatory ones, respectively, with sparse random coupling within and between them (van Vreeswijk and Sompolinsky 1996; Amit and Brunel 1997; Brunel 2000), typically driven by external excitatory input. This architecture is an attractive substrate in which to embed synfire chains because it exhibits a stable state in which neurons spike at a low rate, in an asynchronous irregular (AI) manner which is regarded as a good first approximation to the activity of cortical neurons (Brunel 2000). Next, in order to embed synfire chains into this basic architecture, some or all of the random excitatory connections are replaced with connections that make up the links between successive pools. An upper bound on embedding capacity (the *combinatorial* capacity) is obtained for a given pool size when all the available excitatory connections are replaced with pool-to-pool connections. The smaller the pools, the fewer connections within each pool-to-pool link, and hence the more pools can be embedded (the higher the combinatorial capacity).

Only one published study has systematically investigated the embedding capacity of such systems (Aviel et al. 2005). Like most chain embedding studies to date, Aviel’s study uses current based synapses: each synaptic input generates a fixed-strength pulse of current. The capacity analysis of Aviel et al. assumes the network is in the AI state at a particular mean firing rate and with a characteristic membrane potential distribution. From this the minimum pool size for stable wave propagation is estimated: the minimum number of inputs from one pool to each neuron in the next pool in order for the synfire wave input to shift the membrane potential above threshold with high probability. However, in simulations it was not possible to attain the combinatorial capacity for any pool size. When the fraction of connections used to store pool-to-pool links exceeds a critical level, the AI state spontaneously gives way to a some activity state that prevents synfire propagation. In most of the cases examined, this activity takes the form of strong, periodic bursts of synchronous spikes, but it can also be a state of sustained high firing rate. Even below the critical level for AI state stability, the propagating wave itself drives moderate bursts of synchronized spiking, which tend to destabilize the wave, resulting in a short life time, and the critical level itself is slightly lower in the presence of the wave. The study of Aviel et al. considered the effect of adding inhibitory “shadow” pools to the chains to create a *doubly balanced* architecture in which each pulse packet delivers balanced input to the rest of the network. With double balance, the undesirable effects of the propagating wave are eliminated. As a result, the embedding capacity in a network of *N* neurons is 0.07 *N* pools with double balance as against 0.065 *N* without. In other words, a marginal improvement.^1^

The destabilizing effect of spike packets was also observed in a locally connected recurrent network model representing a small area of cortex with a distance-based Gaussian connectivity density (Mehring et al. 2003). Synchronous activity in a single pool of localized neurons triggers a strong reverberation in the surrounding local region similar to that found by Aviel et al. (2005) even without any embedded chains. Embedding a single chain of 10 pools, the reverberation resulting from the spike packet in the first pool typically extinguishes the synfire wave. Mehring et al. (2003) suggested that the inclusion of inhibitory shadow pools could prevent the reverberation. Surprisingly, even without shadow pools, spike packets can propagate without triggering the reverberation when the current-based synapses are replaced by conductance-based ones, in combination with heterogeneity in neuronal properties including the number of connections per neuron (Kumar et al. 2008). This result was attributed chiefly to the reduction of the local response to the stimulated pulse packet due to the integrative properties of conductance based neurons receiving background input. However, the sources of heterogeneity in the model may also play a role, as previously studies have noted their ability to suppress synchronized oscillations in recurrent networks (Denker et al. 2004; Tetzlaff et al. 2005). Variability in transmission delays has also been suggested as a mechanism to stabilize the AI state (Aviel et al. 2005).

Let us now consider the second requirement for a combinatorial architecture based on synfire chains: the capacity to activate a number of chains simultaneously. Aviel et al. (2005) noted that embedding capacity has a dual nature, involving both the number of available pools and the potential number of co-active waves, but did not incorporate the latter in their analysis. They observed that their system is capable of sustaining only 1 to 3 waves simultaneously. It is known that the storage capacity of sparse patterns in networks of binary neurons drops as the size of the retrieved patterns increases (Tsodyks and Feigelman 1988). Correspondingly, for chain embedding models we may expect that a model with more co-active waves will exhibit a lower embedding capacity, because it operates at a higher overall rate of activity.

A theoretical mean-field analysis (Trengove 2006) predicted embedding capacities of *αN* pools with *α* > 1, much higher than found in previous models using integrate-and-fire dynamics. This was for a model with conductance-based synapses, inhibitory “shadow” pools and variability in the transmission delays on the connections within each pool-to-pool link. However, the study provided no cortical-scale simulations to verify the predictions of the analysis. This is important in order to demonstrate global network stability. With the simulation technology available today this undertaking has become feasible (Morrison et al. 2005).

The present work provides a more thorough mean field analysis of the model of (Trengove 2006) and cortical-scale simulations showing that over a broad range of cortically realistic parameters, global stability is conserved and the mean field analysis holds. The paper is organized as follows. In Section 2 we define the model for cortical embedding of synfire chains: network structure (Section 2.1), neuron and synapse models (Section 2.2). We present the mean field analysis leading to a self-consistency equation relating the parameters (connectivity, pool size) and dynamical variables (firing rates, number of waves) (Section 2.4). We provide a definition of embedding capacity that accommodate its dual nature, involving as it does both the number of pools and the number of co-active waves (Section 2.5). To test the theory, in the simulations we focus on the upper bound on wave activity, attained by ongoing initiation of new waves (Section 2.3). In Section 3 we exhibit typical behaviors of the model (Section 3.1), numerical solutions of key quantities in the mean field analysis (Section 3.2), and compare network behavior to the predictions of the mean field analysis, demonstrating that over a range of cortical parameters the system exhibits the predicted high-capacity (Section 3.4). We give capacity predictions of the mean field analysis as inhibitory synaptic strength is varied, which allow us to specify an optimal inhibitory strength (Section 3.6), and lastly, we compare conductance and current based models using a simplified mean field analysis in order to explain why the former outperforms the latter (Section 3.7). In Section 4 we summarize the results and the reasons why the present model succeeds in attaining a much higher embedding capacity than previous spiking neuron models. We also discuss the modest departures of the simulations from the mean field analysis, the importance of variability in transmission delays and the absence of synaptic rise times in the model, and outline how the present synfire chain embedding model can be extended to encompass cortical embedding of more general structures such as polychronous spiking.

## Methods and models

### Network model for synfire chain embedding

**Fig. 1:**
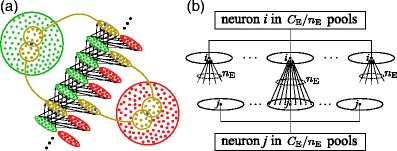
(**a**) Construction of synfire chain embedding in a population of neurons. A sequence of excitatory pools (*green ellipses*) is formed by randomly selecting *n*_E_ distinct neurons from the excitatory population (*large green circle*) *with replacement* to form each pool; two such selections are indicated in *brown*. A corresponding sequence of inhibitory pools (*red*) is formed from the inhibitory population (*red*). Each neuron appears in many pools; e.g. each of the neurons in *blue* appears in two of the pools shown. Links consist of all-to-all connections from each excitatory pool to the next excitatory pool as well as to the corresponding inhibitory pool (*arrows*). (**b**) Origin of excitatory background input. Background input to neuron *i* arises from the activity of the *n*_E_ neurons in each of its *C*_E_/*n*_E_ predecessor pools. Each neuron *j* in a predecessor pool of *i* fires at rate *ν*_S_ + *ν*_W_, with the component *ν*_W_ being due to spike packets in all *C*_E_/*n*_E_ pools that *j* belong to. From this must be subtracted (*n*_E_/*C*_E_)*ν*_W_, the rate due to spike packets in the predecessor pool of *i*. This relationship is formalized in Eq. (4)

The model comprises two populations, *N*_E_ excitatory (E) neurons and *N*_I_ inhibitory (I) neurons respectively. From these constituents, a random superposition of E/I-balanced synfire chains is created as follows. As in previous models, a set of *p* excitatory pools are formed by randomly assigning *n*_E_ excitatory neurons to each pool. Individual neurons can belong to more than one pool: on average a neuron appears in *pn*_E_/*N*_E_ = *αn*_E_ pools, where *α* = *p*/*N*_E_ is the number of pools per neuron, or *embedding level*. We constrained the random assignment so that all neurons appear in nearly the same number of pools. Thus each E-neuron appears in $\left\lfloor p{n_{\mathrm{E}}}/{N_{\mathrm{E}}}\right\rfloor $ or $\left\lceil p{n_{\mathrm{E}}}/{N_{\mathrm{E}}}\right\rceil $ pools, where $\left\lfloor \cdot\right\rfloor $, $\left\lceil \cdot\right\rceil $ denote rounding down, up to the nearest integer. Likewise, *n*_I_ inhibitory neurons are assigned to each of *p* inhibitory (shadow) pools, in 1:1 correspondence with the excitatory ones. Pools can be grouped into sequences to form synfire chains. From a given set of pools, a large number of short chains or a small number of long chains could be formed. Since this makes no difference to the capacity analysis, for convenience we ordered all the E-pools into a single cyclic chain, or ring. Likewise the corresponding I-pools form a shadow ring. Excitatory pool-to-pool links are made by connecting all neurons in each E-pool to all those in the next E-pool and to all those in the next I-pool (see Fig. 1(a)). (We refer to the number of inputs to each neuron in a pool from the previous E-pool as the *input convergence*. Here it equals the pool size, but generally it need not.) Within these constraints the connections are random and sparse; there are no other excitatory connections in the network. Thus the embedding is at the full, combinatorial capacity for the number of connections. Note that the I-pools play no part in wave propagation: their sequential activation is a byproduct of the propagation of a wave down the chain of E-pools. Each E-neuron receives $\left\lfloor p{n_{\mathrm{E}}}/{N_{\mathrm{E}}}\right\rfloor {n_{\mathrm{E}}}$ or $\left\lceil p{n_{\mathrm{E}}}/{N_{\mathrm{E}}}\right\rceil {n_{\mathrm{E}}}$ excitatory connections with

1 $${C_{\mathrm{E}}}=pn_{\mathrm{E}}^{2}/{N_{\mathrm{E}}}=\alpha n_{\mathrm{E}}^{2} $$

being the average number of excitatory inputs per neuron. Each I-neuron receives $\left\lfloor p{n_{\mathrm{I}}}/{N_{\mathrm{I}}}\right\rfloor {n_{\mathrm{E}}}$ or $\left\lceil p{n_{\mathrm{I}}}/{N_{\mathrm{I}}}\right\rceil {n_{\mathrm{E}}}$ excitatory connections. We assume *n*_I_/*n*_E_ = *N*_I_/*N*_E_ = *γ* and use *γ* = 1/4 throughout. This implies that inhibitory neurons appear in just as many pools as excitatory neurons do, and hence the number of excitatory connections received is the same for both types of neurons (*C*_E_). It also ensures that the contribution of each propagating wave to the spiking rate averaged across both populations (E and I) is the same. The inhibitory neurons connect randomly to both populations, subject only to the constraint that all neurons receive the same ratio *γ*′ of inhibitory to excitatory afferents. Hence each neuron in both populations receives inputs from $\gamma'\left\lfloor p{n_{\mathrm{E}}}/{N_{\mathrm{E}}}\right\rfloor {n_{\mathrm{E}}}$ or $\gamma'\left\lceil p{n_{\mathrm{E}}}/{N_{\mathrm{E}}}\right\rceil {n_{\mathrm{E}}}$ randomly selected inhibitory neurons according to how many excitatory inputs it receives, rounded to the nearest integer. Neglecting the small effect of this rounding, the mean number of inhibitory afferents per neuron, *C*_I_, is given by *C*_I_/*C*_E_ = *γ*′. For simplicity we take *γ*′ = *γ*.

### Neuron and synapse model

We use integrate-and-fire neurons with instantaneous conductance-based synaptic responses. Their sub-threshold dynamics is given by:

2 $$\begin{array}{rll} \frac{\mathrm{d} V}{\mathrm{d} t} & = & \frac{{V_{\mathrm{P}}} - V}{{\tau_{\mathrm{P}}}} + \left({V_{\mathrm{E}}} - V\right) \sum_{j,k} g_{\mathrm{E}} \delta\left(t-t_{j}^{k}\right) \nonumber \\ && +\, \left({V_{\mathrm{I}}}-V\right)\sum_{j,k}{g_{\mathrm{I}}}\delta\left(t-t_{j}^{k}\right)\label{eq:sub-thresh-mem-pot-cond-based} \end{array}$$

where *V* is the membrane potential, *V*_P_ is the resting potential, *τ*_P_ is the membrane time constant, *V*_E_ and *V*_I_ are the excitatory and inhibitory reversal potentials, respectively, and *g*_E_ and *g*_I_ are the normalized synaptic conductance responses to input, respectively (the same for all synapses of each type).^2^ A spike is emitted when *V* = *V*_Θ_ and the membrane potential is reset to *V* = *V*_R_, where *V*_Θ_ and *V*_R_ are threshold and reset values, respectively. The membrane potential remains at *V*_R_ for a refractory period, *τ*_ref_. Table 1 gives the values of these parameters that were used in the chain embedding simulations and, except where otherwise noted, elsewhere.

**Table 1:** Neuronal and synaptic parameter values

*V*_E_	0	mV
*V*_I_	− 80	mV
*V*_P_	− 70	mV
*V*_R_	− 70	mV
*V*_Θ_	− 55	mV
*τ*_P_	20	ms
*τ*_ref_	2	ms
*g*_E_	0.005	–
*g*_I_	0.11	–

Transmission delays vary across individual synaptic connections. Identical delays are unrealistic and, moreover, in a model with instantaneous synaptic responses, identical delays would allow the propagation of perfectly synchronized spike packets; there would then be no interval after the arrival of the synchronous inputs during which background input could prevent or modulate the timing of an output spike. Variability of delays introduces such an interval. As a consequence, the model does not require rise times in the post synaptic potentials, which would provide an alternative means for creating such an interval.

The excitatory delays were specified so as to give a realistically broad delay distribution overall, but a narrow distribution of the delays on the synapses from any one pool to the next pool in the sequence. To achieve these two properties, each synapse’s transmission delay was set to be the sum of two components: a component with large variability which is the same for all synapses within the same pool-to-pool link (*τ*_A_); and a component with small variability which is different for every synapse (*τ*_B_). Thus the transmission delay on the synaptic connection from the *j*th neuron in the *μ*th E-pool to the *i*th neuron in the next E/I-pool is given by *τ*_A_(*μ*) + *τ*_B_(*μ*,*i*,*j*) where *τ*_A_(*μ*) is drawn from a uniform distribution on $\left[0.5,4.5\right)$ ms and *τ*_B_(*μ*,*i*,*j*) is drawn from a uniform distribution on $\left[0,0.5\right)$ ms. This specification is complete and self-consistent because every excitatory synapse belongs to exactly one pool-to-pool link. We refer to *τ*_A_ as the inter-link delay, with a spread of 4.0 ms, and *τ*_B_ as the intra-link delay, with a spread of 0.5 ms.

The relatively small variability of delays within synfire links is a defining feature of synfire chains: propagation by near-synchronous input. It is also plausible if, as argued by Bienenstock (1991, 1995), synfire chains develop by strengthening synapses that cooperate to deliver synchronous inputs. The magnitude and variability of the transmission delays are realistic for intracortical connectivity on the scale of a few hundred millimeters given typically observed intracortical conduction velocities in the range of 0.1–0.5 m/s (Yger et al. 2011; Bringuier et al. 1999; González-Burgos et al. 2000). This is around the low end of the velocity range observed for cortico-cortical connections (Swadlow 2000). For instance, a synaptic delay of 0.5 ms, an axonal path length of 400 μm, and a velocity of 0.2 m/s would reproduce the mean transmission delay of 2.5 ms used here. The inhibitory connection delays vary according to the same two-component distribution, but in their case both components are drawn independently for every synapse.

### External input

As opposed to most balanced random architecture models, our network does *not* receive steady, ongoing excitatory input, neither continuous current nor a stochastic (Poisson) input at a constant rate. The model thereby demonstrates that synfire waves alone can drive ongoing activity in a network. By omitting ongoing external excitatory drive, the ratio of excitatory to inhibitory background input becomes invariant with respect to the mean firing rate of the network (see Section 2.4).

**Fig. 2:**
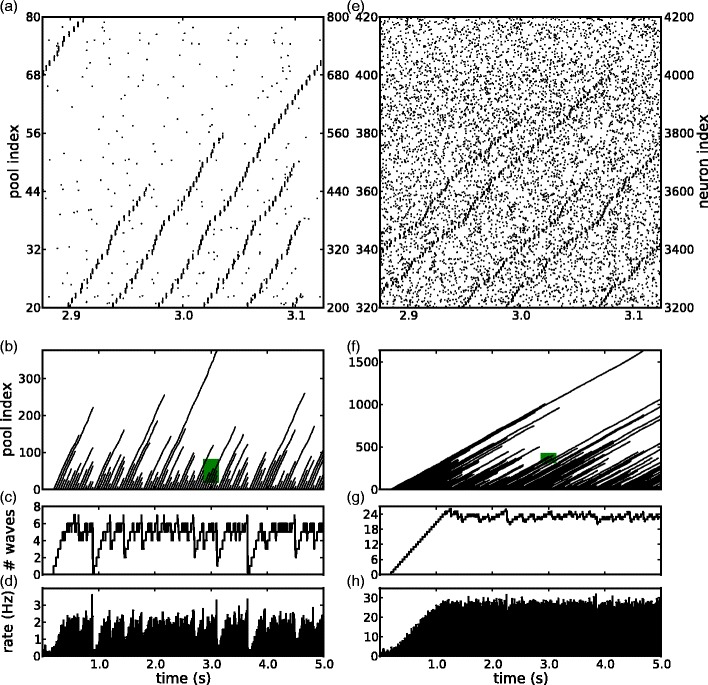
Wave activity and mean spiking rate in networks with ${C_{\mathrm{E}}}=8\text{,}000$; (a-d) small pools, *n*_E_ = 72; (**e**–**h**) large pools, *n*_E_ = 200 (**a**,**e**) Spike rasters of neurons in (**a**) pools 20 to 79, (**e**) pools 320 to 419 in the sequence beginning with the stimulated pool (*left axis index*). For each pool, ten arbitrarily chosen neurons are shown (indexed cumulatively on the *right axis*); (**b**,**f**) raster of extracted spike packets, rectangles indicate regions shown in (**a**,**e**); (**c**,**g**) number of waves; (**d**,**h**) population mean rate histograms

External input to the network is in the form of near-synchronous packets of excitatory input to a specific E-pool (and its corresponding I-pool) that serve to initiate synfire waves. Each such stimulus, occurring at a given time *t*, consisted of *n*_E_ spikes normally distributed in time with mean *t* and standard deviation 0.1 ms, which were delivered to each neuron in the target pool, with a different transmission delay for each spike, these delays being drawn from the same distribution as the intra-link delay component *τ*_B_ (Section 1).^3^ These stimuli are ongoing, in order to drive the number of synfire waves to a maximum, and to demonstrate that the system remains globally stable in such conditions. The stimuli are regularly spaced at 40 ms intervals from *t* = 200 ms onwards. For convenience of output display (Fig. 2) we always stimulated the same pool but the behavior will be essentially the same whatever the sequence of stimulated pools, provided the resulting waves do not interfere with each other by visiting the same pools in close temporal succession.

The first four wave-initiating stimuli are accompanied by a transient of E/I balanced external Poisson input. This begins at time zero with E/I rates approximately equal to the background input rates generated by 4 synfire waves. The rates are progressively reduced to zero in four equally-sized steps, one at each new stimulus. This was done to ensure that the mean membrane potential is near equilibrium when the first four stimuli arrive, rather than being at the resting potential, in case the latter would be too low to allow a spike packet to initiate a wave. It proved not essential, however, for the working of the model.

### Mean field analysis

We begin by analyzing the equilibrium state of a network in which there is assumed to be a fixed number of propagating synfire waves, *h*, but no external stimulation of new waves such as specified in Section 2.3, nor any other external input.^4^ A mean field analysis can be formulated in which the equilibrium state is characterized by a single global parameter, the population-averaged mean firing rate. The rate, as we will see, is the same for both excitatory and inhibitory populations. It can be decomposed into two parts:

3 $$ \nu={\nu_{\mathrm{W}}}+{\nu_{\mathrm{S}}}\label{eq:nu} $$

with *ν*_W_ being the mean rate of spikes belonging to synfire waves and *ν*_S_ being the mean rate of the remaining spikes. These are what we referred to in Section 1 as stochastic spikes, because they arise from stochastic membrane potential fluctuations driven by background input*. *Background input refers to all the inputs to a neuron, other than the synchronous excitatory inputs from a synfire wave. Asynchronous input from excitatory and inhibitory neurons, respectively, arrives at rates

4 $${\lambda_{\mathrm{E}}}={C_{\mathrm{E}}}\left({\nu_{\mathrm{W}}}\left(1-{n_{\mathrm{E}}}/{C_{\mathrm{E}}}\right)+{\nu_{\mathrm{S}}}\right)\approx{C_{\mathrm{E}}}\nu\label{eq:lambdaE} $$

and

5 $${\lambda_{\mathrm{I}}}={C_{\mathrm{I}}}\nu\,.\label{eq:lambdaI} $$

Figure 1(b) illustrates how the *excitatory* background input arises. Consider one of the neurons contributing to the background input. Each such neuron belongs to *C*_E_/*n*_E_ pools, and is thus visited by waves at different times, resulting in spikes at quasi-random intervals, at a mean rate *ν*_W_ over the population. In addition, each neuron has a component of stochastic spiking at rate *ν*_S_. For the excitatory inputs to a neuron *i*, this leads to a rate *C*_E_(*ν*_W_ + *ν*_S_) − *n*_E_ *ν*_W_, based on the activity in the predecessor pools of all the pools to which *i* belongs, minus the fraction due to synchronous activity in each of these predecessor pools.^5^ Since this fraction, *n*_E_/(*C*_E_(1 + *ν*_S_/*ν*_W_)), is only small in our model, Eq. (4) provides a good approximation. This is convenient because it renders *λ*_I_/*λ*_E_ = *C*_I_/*C*_E_ = *γ*, independently of *C*_E_ and *n*_E_. The *C*_I_ inhibitory inputs per neuron are uncorrelated with synfire links, and therefore the rate in Eq. (5) is exact.

Background input does two things. First, it generates stochastic spiking at rate *ν*_S_. Second, it modulates spike packet propagation, and thus the rate of spikes participating in synfire waves, *ν*_W_. These effects have to be combined with those whereby the spiking rate in turn generates the background input (Eqs. (3)–(5)), in order to obtain a self-consistent description that determines the steady state behaviour for a given number of propagating waves.

The stochastic spiking rate is approximated as the mean spike rate of a single neuron receiving Poisson input streams of excitatory and inhibitory inputs at rates *λ*_E_ and *λ*_I_ respectively, denoted by the function

6 $${\nu_{\mathrm{S}}}=f_{\mathrm{S}}({\lambda_{\mathrm{E}}},{\lambda_{\mathrm{I}}})\,.\label{eq:nuS} $$

There is an analytical expression for the function *f*_S_, the so-called Siegert formula (Burkitt 2006), which we use to compare current-based and conductance-based models in Section 3.7. However, this expression can be a poor approximation if the conductances are not small enough and, in a time-step based simulation, if the time-step is not small enough. Hence, for accurate comparison of network simulations and mean field theory, the function *f*_S_ was computed by single neuron simulations for a closely spaced sequence of *λ*_E_ values (with *λ*_I_ = *γλ*_E_) and extended to intermediate values of *λ*_E_ by linear interpolation. The single neuron simulations used a 0.1 ms time step, same as for the network. Each simulation consisted of a set of 100 runs of duration of 5,000 ms. The mean spike rate over the interval $[1\text{,}000\mathrm{\, ms},\,5\text{,}000\,\mathrm{ms}]$ was computed. This procedure was sufficient to obtain an accurate estimate of the spiking rate.

The modulation of spike-packet propagation by background input was characterized via numerical simulations of a non-embedded chain of non-overlapping pools where each neuron receives external, uncorrelated Poisson streams of input at rates *λ*_E_ and *λ*_I_ = *γλ*_E_ respectively. These simulations were done for pool sizes ${n_{\mathrm{E}}}\in\left\{ 20,24,\ldots,220\right\} $ and rates, ${\lambda_{\mathrm{E}}}\in\left\{ 1,2,\ldots,300\right\} $ kHz using a 0.1 ms time step. For each choice of pool size and background input rate, a chain of 100 E-pools was created with the same intra-link delay distribution as in the chain embedding model (*τ*_B_ in Section 2.2). Each simulation began with a period of 100 ms in which only background input was present. This was done to avoid onset transients. A wave-initiating excitatory input was then delivered to the third pool in the chain as follows: *n*_E_ spikes, normally distributed in time with mean 100 ms and standard deviation 0.1 ms, were transmitted according to the same delay distribution as if they had occurred in the predecessor pool. The probability *P*_S_(*λ*_E_,*λ*_I_;*n*_E_) that a wave would successfully propagate to the end of the chain (that is, over 98 pools) was estimated by obtaining, over 100 trials, the fraction in which a spike packet was detected in the last pool (see Section 2.6). From this we calculated the average size of spike packets over successful wave propagation trials. The average spike-packet size was normalized by dividing by *n*_E_, to give *p*_f_(*λ*_E_,*λ*_I_), essentially the probability that any given neuron in the pool contributes a spike to the packet. The mean time interval between the spike packets in the 90th and 100th pools, divided by 10, was used to estimate *T*(*λ*_E_,*λ*_I_), the mean pool-to-pool propagation time. The rate of wave spiking in the chain embedding network is then given by

7 $${\nu_{\mathrm{W}}}=f_{\mathrm{W}}({\lambda_{\mathrm{E}}},{\lambda_{\mathrm{I}}},h,{n_{\mathrm{E}}})=\frac{h{n_{\mathrm{E}}}{p_{\mathrm{f}}}({\lambda_{\mathrm{E}}},{\lambda_{\mathrm{I}}})}{{N_{\mathrm{E}}} T({\lambda_{\mathrm{E}}},{\lambda_{\mathrm{I}}})}\label{eq:fW} $$

Equations (3)–(7) determine the mean firing rate, *ν*, along with the background input rates and the rates of wave spiking and stochastic spiking for a given number of waves, *h*. Since *f*_W_ and *f*_S_ are obtained via numerical simulations, it is convenient that *λ*_I_/*λ*_E_ is an invariant of the dynamics (*λ*_I_/*λ*_E_ = *γ*), since it allows *f*_S_, *p*_f_ and *T* to be reduced to functions of one variable (*λ*_E_). Equations (3)–(7) then lead to a single equation for the excitatory background input rate, and hence the other rates (*λ*_I_, *ν*, *ν*_W_, *ν*_S_):

8 $$\begin{array}{rll} {\lambda_{\mathrm{E}}} & = & {C_{\mathrm{E}}}\left[\left(h/{N_{\mathrm{E}}}\right){n_{\mathrm{E}}}{p_{\mathrm{f}}}({\lambda_{\mathrm{E}}},\gamma{\lambda_{\mathrm{E}}})/T({\lambda_{\mathrm{E}}},\gamma{\lambda_{\mathrm{E}}}) \right. \nonumber \\ && \left. +f_{\mathrm{S}}({\lambda_{\mathrm{E}}},\gamma{\lambda_{\mathrm{E}}})\right]\label{eq:self-consistent-rate} \end{array}$$

This shows that *C*_E_, *h*/*N*_E_, and *n*_E_ determine the stationary rates according to the mean field analysis. Alternatively, since ${C_{\mathrm{E}}}=\left(p/{N_{\mathrm{E}}}\right)n_{\mathrm{E}}^{2}$ the stationary rates are determined by *p*/*N*_E_, *h*/*N*_E_, and *n*_E_. This implies that for a given pool size *n*_E_, the behaviour is invariant if both the number of pools and the number of waves scale linearly with *N*_E_. In practice we choose*N*_E_ = 10*C*_E_ to obtain a network with 10 % probability of connection for any pair of neurons.

### Embedding capacity

We would like a measure of embedding capacity to allow comparison across ranges of system parameters; in particular, connectivity and inhibitory conductance strength. Embedding capacity is the largest embedding level that the system can incorporate while functioning properly; i.e. allowing stable propagation of a certain number of waves and a stability of the global rate dynamics.

First, the mean field analysis of Section 2.4 must be extended to include the constraint of stable wave propagation, which is assumed in obtaining the steady state solution given by Eq. (8). For spike packet propagation to be stable there will, for a given synaptic strength and level of background input, be a minimum pool size (Diesmann et al. 1999). Using the function *P*_S_(*λ*_E_,*γλ*_E_;*n*_E_) that specifies the probability of survival, we identify the minimum pool size, *n*_E,min_(*λ*_E_), by the condition

9 $$ P_{\mathrm{S}}({\lambda_{\mathrm{E}}},\gamma{\lambda_{\mathrm{E}}};{n_{\mathrm{E}}})>0.5\Longleftrightarrow{n_{\mathrm{E}}}>n_{\mathrm{E,min}}({\lambda_{\mathrm{E}}}). $$

The steady state solution specified by Eq. (8) for an arbitrary number of waves will be at the capacity limit if we add the condition *n*_E_ = *n*_E,min_(*λ*_E_).

This then allows us to define the capacity limit of the system as a constraint on both the number of pools embedded and the level of activity, taken jointly. In particular, if we specify the level of activity in terms of the mean firing rate, *ν*, then setting *n*_E_ = *n*_E,min_(*C*_E_ *ν*) gives, as a function of the connectivity *C*_E_, the largest embedding level ($\alpha=C_{\rm {E}}/n_{\rm {E}}^{2}$) that allows stable propagation for a global mean firing rate *ν*. That is

10 $${\alpha_{\mathrm{max}}}({C_{\mathrm{E}}};\nu)={C_{\mathrm{E}}}/n_{\mathrm{E,min}}^{2}({C_{\mathrm{E}}}\nu) $$

Alternatively, we can specify the level of activity in terms of the relative number of waves, *h*/*N*_E_, and obtain a *different* embedding capacity function of connectivity, *α*_max_(*C*_E_;*h*/*N*_E_). For each of these definitions of *α*_max_(*C*_E_), we optimize embedding capacity with respect to connectivity.

The connectivity range is constrained by the requirement that there is a stable steady state level of stochastic spiking activity in the system. Here we use a “naive” estimate of the stability of stochastic spiking in terms of the equivalent balanced random network. A given self-consistent solution of the mean field theory with mean rates *ν*, *ν*_W_ and *ν*_S_ satisfies ${\nu_{\mathrm{S}}}=\hat{f}_{\mathrm{S}}({C_{\mathrm{E}}}({\nu_{\mathrm{W}}}+{\nu_{\mathrm{S}}}))$ where ${\nu_{\mathrm{S}}}={\hat{f}_{\mathrm{S}}}({\lambda_{\mathrm{E}}})\equiv{f_{\mathrm{S}}}({\lambda_{\mathrm{E}}},\gamma{\lambda_{\mathrm{I}}})$. Being the fixed point of the mapping $\nu_{\mathrm{S}}^{\mathrm{out}}=\hat{f}_{\mathrm{S}}({C_{\mathrm{E}}}({\nu_{\mathrm{W}}}+\nu_{\mathrm{S}}^{\mathrm{in}}))$, the solution is considered stable if the condition

11 $$ \left|\left.\partial\hat{f}_{\mathrm{S}}\left({C_{\mathrm{E}}}\left({\nu_{\mathrm{W}}}+\nu_{\mathrm{S}}^{\mathrm{in}}\right)\right)/\partial\nu_{S}^{\mathrm{in}}\right|_{\nu_{\mathrm{S}}^{\mathrm{in}}={\nu_{\mathrm{S}}}}\right|<1\label{eq:nuS_stability} $$

holds.^6^ Because $\hat{f_{\mathrm{S}}}$ increases at a roughly constant rate while well below saturation, this leads to an upper limit on connectivity:

12 $${C_{\mathrm{E}}}<{C_{\mathrm{E,max1}}}= 1/\left.\left(\mathrm{d}\hat{f_{\mathrm{S}}}/\mathrm{d}{\lambda_{\mathrm{E}}}\right)\right|_{{\lambda_{\mathrm{E}}}={C_{\mathrm{E}}}\nu}. $$

Even below the limit for stochastic spike rate stability, it is necessary to ensure that a reasonable amount of wave activity is present at the capacity limit. As *C*_E_ increases, holding *ν* = *ν*_W_ + *ν*_S_ constant, the level of stochastic spiking determined by ${\nu_{\mathrm{S}}}=\hat{f}_{\mathrm{S}}({C_{\mathrm{E}}}({\nu_{\mathrm{W}}}+{\nu_{\mathrm{S}}}))$ will increase, while the fraction of spikes due to synfire waves will decrease. We therefore also require that at least 50 % of the spiking should belong to wave activity, which leads to another (usually smaller) connectivity limit, *C*_E,max2_.

Since the embedded chain network is *not* a random network, we rely on simulations to verify both the accuracy and the stability of the mean field solution for the system at capacity, when the connectivity is below both these limits, *C*_E,max1_ and *C*_E,max2_.

### Detection of spike packet sequences

Detecting unitary events from a stream of activity can be done in a variety of ways. Our method is suitable for situations where the spike rate outside a spike packet is low; thus we can use a detection window wide enough to safely cover any typical spike packet while including a negligible number of non-packet spikes. Within such windows, the median spike time gives the precise location of spike packet. We combined all the spike trains of all neurons in the pool to obtain a list of spiking times *T*_*s*_ = *t*_1_,...,*t*_*n*_. To every spike time *t*_*k*_ in *T*_*s*_, a window [*t*_*k*_,*t*_*k*_ + *T*_*w*_) was applied, with width *T*_*w*_ = 3 ms. The spike times within this window constitute a sublist *S*_*k*_. Sublists were classified as *suprathreshold* if they contained more than *n*_*θ*_ spike times (|*S*_*k*_| > *n*_*θ*_). *n*_*θ*_ was set to a value midway between an estimated minimum spike packet size and an estimated maximum background spike count, resulting typically in *n*_*θ*_ ≈ 0.4*n*_E_. Maximal consecutive sequences of at least 6 suprathreshold sublists were identified; that is, sequences of the form *L* = (*S*_*k* + 1_,...,*S*_*l*_) where *l* − *k* ≥ 6, |*S*_*j*_| > *n*_*θ*_ for *j* ∈ *k* + 1,...,*l*, |*S*_*k*_| ≤ *n*_*θ*_ and |*S*_*l* + 1_| ≤ *n*_*θ*_. The minimum sequence length, 6, was chosen to exclude small suprathreshold sequences that can arise when |*S*_*j*_| ≈ *n*_*θ*_ due to fluctuations in successive |*S*_*j*_|. Each such sequence *L* determines a single spike packet, as follows. We identify amongst the sublists in the sequence the ones with maximum activity: *L*_max_ = (*S*_*j*_ ∈ *L* : |*S*_*j*_| =  max {|*S*_*i*_| : *S*_*i*_ ∈ *L*}). We identify their “middle”, the $\left\lfloor |L_{\max}|/2\right\rfloor $’th sublist in *L*_ max _, as the spike packet. The time of the spike packet was defined as the median of its spike times. From the extracted spike packets we compute *ν*_W_(*t*), a time-resolved mean rate over the population of spikes belonging to spike packets, using a bin size of 20 ms. By linking up spike packets on consecutive pools, synfire waves were identified. Spike packets on two consecutive pools were linked up if the time interval between the first and second was between 0.5 and 6 ms.

### Simulation tools

Simulations of the chain embedding networks were computationally intensive due to the large size of the networks. We used the NEST simulation software using the Message Passing Protocol (MPI) for parallel processing (Morrison et al. 2005), running on a cluster of 24 Linux PCs with a total of 192 cores and 192 GB of memory. The largest networks we simulated (those with ${C_{\mathrm{E}}}=11\text{,}000$) had approximately 1.4×10^5^ neurons and 1.9×10^9^ synapses, which is close to the maximum possible with these resources.

## Results

### Behavior of large scale network simulations

Figure 2 exhibits the behavior of two networks, each with connectivity per neuron ${C_{\mathrm{E}}}=8\text{,}000$. In both cases, the model exhibits stable activity, and shows the emergence of an upper bound on the number of simultaneously propagating spike-packets. In the left side of Fig. 2, pool size is near the low end for allowing viable wave propagation; this results in a high embedding level and a large number of pools (*n*_E_ = 72, *α* = 1.54, *p* = 123457). In the right side of the figure, pool size is about 3 times larger; this leads to a lower embedding level and a smaller number of pools, (*n*_E_ = 200, *α* = 0.2, $p=16\text{,}000$). Note that even in this case, the number of pools is still high compared to previous studies. Figure 2(a) and (e) give spike rasters for 10 neurons from each pool in a sequence of consecutive pools. The vertical axis indexes pools counted from the stimulated pool (left) and the cumulative count of neurons plotted (right). Since each neuron appears in many pools, its spike train is plotted at many vertical positions. Within a pool, spikes belonging to spike packets are visibly distinguishable from the remaining ’background’ spikes. As discussed in Section 2.4, these are due partly to stochastic spiking, and partly due to the neurons participating in the spike packets of other pools. The raster plots clearly show waves of spike packets, propagating as a consequence of the 25 Hz periodic stimulation. The raster plots of *all *extracted spike packets (Fig. 2(b) and (f)) verify that all spike packets belong to waves that propagate down the chain from the stimulated pool; none occur spontaneously or out of sequence. In the traces of how the number of co-active waves varies over time (Fig. 2(c) and (g)), for both cases, the number of waves fluctuates around a mean value not far below the maximum. With small-sized pools, the number of waves occasionally drops abruptly to zero or a small fraction of the maximum, from which it gradually recovers as new waves are injected every 40 ms. In contrast, for large-sized pools the number of waves remains relatively close to the mean. This is because with smaller pools there are fewer waves and their survival is more affected by the noise feedback: they have a shorter average life time indicating that they are less robust, as discussed in Section 3.3. The mean firing rate in the population versus time (Fig. 2(d) and (h)) closely tracks the number of waves simultaneously present. With many, small-sized pools, the upper bound is 7 waves (with a mean of 4.9 and a firing rate of 1.75 Hz), while with fewer but larger pools it is 26 waves (with a mean of 23.0 and a firing rate of 26.3 Hz). This apparent trade-off between the number of pools and the maximum number of co-active waves is described systematically in Section 3.4.

### Dependence of pulse-packet propagation properties and stochastic spiking on background input

**Fig. 3:**
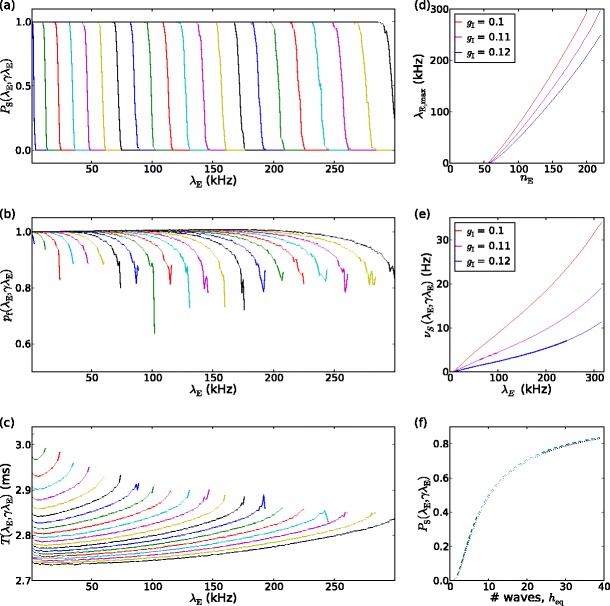
Dependence of wave propagation and stochastic spiking on background input rate, *λ*_E_. (**a**) probability of wave propagation over 98 pools, *P*_S_(*λ*_E_,*γλ*_E_), for *λ*_E_ ∈ [0,300] kHz and pool sizes *n*_E_ ∈ 60,68,...,220; *λ*_E,max_: *P*_S_(*λ*_E,max_,*γλ*_E,max_) = 0.5 increases with *n*_E_. (**b**) probability that each neuron in a pool participates in a spike packet, *p*_f_, and (**c**) mean pool-to-pool propagation time of a wave, *T*, for *n*_E_ ∈ 60,68,...,220; in (**b**,**c**) each *curve* is defined on for *λ*_E_: *P*_S_(*λ*_E_,*γλ*_E_) > 0, the upper bound of which increases with *n*_E_. (**d**) maximum background input rate for propagation versus pool size, *λ*_E,max_(*n*_E_). (**e**) stochastic spiking rate *ν*_S_ = *f*_S_(*λ*_E_,*γλ*_E_) for three values of inhibitory synaptic conductance, *g*_I_. (**f**) probability of wave survival over 98 pools versus equilibrium number of waves when driven by external wave stimulation at rate 25 Hz for *n*_E_ = 80 (*dotted*), *n*_E_ = 200 (*dashed*). (See text, Section 3.3)

Here we give the numerical solutions for quantities required for the mean field analysis of Section 2.4. Figure 3(a) show *P*_S_(*λ*_E_,*γλ*_E_), the probability of a synfire wave propagating over a chain of 98 pools, as a function of background input rate *λ*_E_ for pool sizes *n*_E_ ∈ 60,68,...,220. For *n*_E_ < 60, *P*_S_ = 0 over the range of *λ*_E_ examined, while *P*_S_ = 1 for *n*_E_ > 224. Between these extremes, *P*_S_ has as a sigmoidal form: the probability of successful propagation is high for sufficiently low values of *λ*_E_ and drops rapidly to zero when background input rate reaches a critical level. We can therefore define a threshold rate for successful propagation, *λ*_E_ = *λ*_E,max_:

13 $${\lambda_{\mathrm{E,max}}}({n_{\mathrm{E}}})={\lambda_{\mathrm{E}}}\,:\,P_{\mathrm{S}}({\lambda_{\mathrm{E}}},\gamma{\lambda_{\mathrm{E}}};{n_{\mathrm{E}}})=0.5\label{eq:lambdaEmax} $$

This threshold increases with pool size, as shown in Fig. 3(d) for 3 values of inhibitory conductance (*g*_I_ = 0.1, 0.11, 0.12). Increasing the inhibitory conductance reduces the threshold. Conversely, this relationship defines a minimum pool size for propagation at a given background input rate: ${n_{\mathrm{E}}}=n_{\mathrm{E,min}}({\lambda_{\mathrm{E}}})\Leftrightarrow{\lambda_{\mathrm{E}}}={\lambda_{\mathrm{E,max}}}({n_{\mathrm{E}}})$.

Note that the existence of a threshold rate above which propagation fails is non-trivial. It is to be expected that there will be a minimum pool size for propagation at a given background rate, but only if *n*_E,min_(*λ*_E_) is an increasing function at a given rate *λ*_E_ will that rate *λ*_E_ be an upper limit for propagation over pools of size *n*_E,min_(*λ*_E_). The important functional consequence is that background input serves to regulate wave activity; since waves generate background input, with ongoing wave-initiating stimuli the number of waves will increase until background rate reaches the threshold, as seen in Fig. 2. The system equilibrates at the embedding capacity limit where *n*_E_ = *n*_E,min_(*λ*_E_).

The probability that a neuron in a pool participates in a given spike packet in that pool, *p*_f_(*λ*_E_,*γλ*_E_) and the mean pool-to-pool propagation time *T*(*λ*_E_,*γλ*_E_) are given in Fig. 3(b, c) respectively. *p*_f_ and *T* depend relatively weakly on background input except when *λ*_E_ exceeds the threshold level. Figure 3(e) shows, for the 3 values of *g*_I_ given in Table 1, *f*_S_(*λ*_E_,*γλ*_E_), the mean spiking rate of a neuron receiving balanced background input. The rate is approximately linear in *λ*_E_ with a slope that depends strongly on ratio of the synaptic conductances, *g*_E_/*g*_I_.

### Regulation of wave activity: the equilibrium state of the driven system

Figure 3(a) and (d) demonstrated the existence of a threshold level of background input (*λ*_E,max_ in Eq. (13)), around which wave survival probability rapidly drops from 1 to 0. This threshold is responsible for the upper bound on the number of waves seen in Fig. 2. Background input is generated as a by-product of the waves (Eq. (8)). Thus, as the number of waves increases with ongoing stimulation, at the same time the background input rises until it reaches the vicinity of the threshold. At this point, the survival time of waves rapidly drops until an equilibrium point is reached, where the wave initiation rate is counterbalanced by the rate of wave failure.

The number of waves at which* equilibration* occurs can be quantified by considering the expected lifetime of a wave, denoted *T*_S_(*λ*_E_).^7^ As a wave progresses along the chain, the expected probability of failure per step is constant for a given background rate and independent of previous steps. We may therefore assume that the life times of waves obey an exponential distribution (as observed in the model by Bienenstock 1995). Then *T*_S_(*λ*_E_) is related to *P*_S_, the probability of survival over *L* = 98 pools, by:

14 $$ T_{\mathrm{S}}({\lambda_{\mathrm{E}}})=T({\lambda_{\mathrm{E}}})L/\log(1/P_{\mathrm{S}}({\lambda_{\mathrm{E}}}))\label{eq:TS} $$

with *L*/log(1/*P*_S_) being the expected number of pools traversed. In the system driven by external stimulation, the number of waves reaches equilibrium when the death rate equals the rate of wave stimulation rate, so the expected life time is related to the equilibrium number of waves, *h*_eq_, and the stimulation period, *T*_stim_, by *h*_eq_ = *T*_S_/*T*_stim_. The equilibrium number of waves is thus related to the probability of survival over *L* pools by:

15 $$ h_{\mathrm{eq}}=T({\lambda_{\mathrm{E}}})L/\left(T_{\mathrm{stim}}\log(1/P_{\mathrm{S}}({\lambda_{\mathrm{E}}}))\right)\label{eq:h-Ps} $$

or, equivalently

16 $$ P_{\mathrm{S}}=\exp\left(\frac{-LT({\lambda_{\mathrm{E}}})}{h_{\mathrm{eq}}T_{\mathrm{stim}}}\right)\approx\exp\left(-L\hat{T}/h_{\mathrm{eq}}T_{\mathrm{stim}}\right) $$

where the weak *λ*_E_-dependence of *T*(*λ*_E_) and the steep variation of *P*_S_(*λ*_E_) for *λ*_E_ ≈ *λ*_E,max_ allows *T*(*λ*_E_) to be replaced by $\hat{T}\equiv T({\lambda_{\mathrm{E,max}}}({n_{\mathrm{E}}}))$. This approximate relationship between probability of survival and mean number of waves is illustrated in Fig. 3(f) for *n*_E_ = 80 and *n*_E_ = 200. It depends only very weakly on *n*_E_. Figure 3(f) shows that there is a range of possible equilibrium numbers of waves according to the value of *P*_S_(*λ*_E_). These occur at *λ*_E_-values close to *λ*_E,max_, since *P*_S_(*λ*_E_) has a sigmoidal form that sharply drops from 1 to 0. An expression for the equilibrium background input rate *λ*_E_, can be obtained by combining Eqs. (3), (4), (7), (6) and (15):

17 $$ \frac{L{n_{\mathrm{E}}}{p_{\mathrm{f}}}({\lambda_{\mathrm{E}}})}{T_{\mathrm{stim}}\log\left(1/P_{\mathrm{S}}({\lambda_{\mathrm{E}}})\right){N_{\mathrm{E}}}}={\lambda_{\mathrm{E}}}/{C_{\mathrm{E}}}-f_{\mathrm{S}}({\lambda_{\mathrm{E}}}) $$

The mean spike rates *ν*_W_, *ν*_S_ and *ν* at equilibrium and the equilibrium number of waves *h*_eq_ are then determined by using this value of *λ*_E_ in Eqs. (7), (6), (3) and (15), respectively.

In practice, however, using *λ*_E_ = *λ*_E,max_ to evaluate *ν*_W_, *ν*_S_, *ν* and *h*_eq_ is a good enough approximation because of the sharp transition of *P*_S_ from 1 to 0. This is the method used to define the embedding capacity limit in Section 2.5.

### Comparison of embedding capacity limit by analysis and by network simulations

**Fig. 4:**
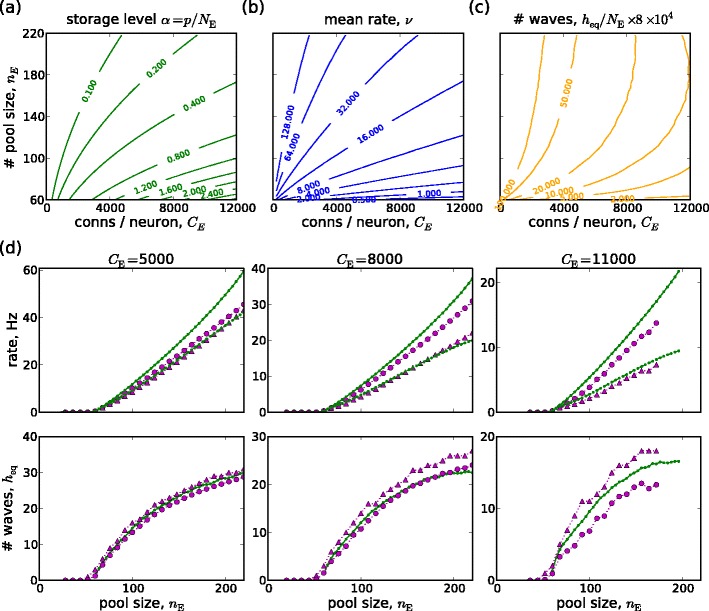
(**a**–**c**) Predictions of mean field analysis for embedding level (*α* = *p*/*N*_E_), mean firing rate (*ν*) and equilibrium number of synfire waves per neuron (*h*_eq_/*N*_E_) as functions of pool size and connectivity. (**d**) comparison between mean field analysis and simulations for connectivity ${C_{\mathrm{E}}}={5\text{,}000,8\text{,}000,11\text{,}000}$. For each *C*_E_: *upper plot*, overall mean rate and mean rate due to wave spiking versus pool size for the mean field solution (*small green markers*) and for simulations (*large magenta markers*); *lower plot*, versus pool size, the mean field prediction for the equilibrium number of waves (*small green markers*) and the maximum and mean numbers of waves in the simulations (*large magenta triangles* and *circles*, respectively)

We have seen that the system driven by external wave stimuli equilibrates at the embedding capacity limit. Figure 4 shows how the state of the system at the embedding capacity limit depends on connectivity and pool size, as predicted by the mean field analysis. Figure 4(a) gives the embedding level ($\alpha=p/{N_{\mathrm{E}}}={C_{\mathrm{E}}}/n_{\mathrm{E}}^{2}$). Figure 4(b) and (c) show, respectively, the mean firing rate at the limit, and the equilibrium number of waves (*h*_eq_). The plots reveal a systematic trade-off between on the one hand, the number of waves and mean firing rate, and, on the other, the embedding level. Smaller pools and higher connectivity per neuron directly specify a higher embedding level ($\alpha={C_{\mathrm{E}}}/n_{\mathrm{E}}^{2}$) and result in lower firing rates and lower numbers of waves, and vice versa.

We compared the equilibrium firing rates and numbers of waves predicted by the mean field analysis with those found in a series of simulated networks, for connectivity ${C_{\mathrm{E}}}\in{5\text{,}000,6\text{,}000,\ldots,11\text{,}000}$ and pool size *n*_E_ ∈ 20,28,...,220. For each choice of *C*_E_ and *n*_E_, we conducted one network simulation of 10,000 ms duration, extracted spike packets sequences (Section 2.6) and obtained time resolved mean spiking rates *ν*(*t*), *ν*_W_(*t*) and number of waves *h*(*t*). An initial transient, the time taken for the number of waves to reach equilibrium, was excluded. We set $t_{\mathrm{start}}=\max(1\text{,}000\,\mathrm{ms},\, t_{\mathrm{mean}})$ where *t*_mean_ is the time when *h*(*t*) first exceeds the mean over the interval [1,000 ms, 10,000 ms]. The mean rate of all spiking activity (*ν*), the mean rate of spikes belonging to spike packets (*ν*_W_) and the mean and maximum number of waves were then determined over the period $[t_{\mathrm{start}},\,10\text{,}000\,\mathrm{ms}]$.

The model reaches stable equilibria in all but a few cases. The few exceptions occurred when both pool size and connectivity was large (*n*_E_ = 212,220 for ${C_{\mathrm{E}}}=10\text{,}000$ and *n*_E_ = 204,212,220 for ${C_{\mathrm{E}}}=11\text{,}000$). In these five cases, network behaviour was “normal” until some apparently arbitrary point at which it rose very rapidly to the saturation rate imposed by the neuronal refractory period. The rate explosion is of course incompatible with wave further propagation. No spontaneous waves were observed to appear before the rate explosion occurred.

Figure 4(d) shows the equilibrium behavior for both simulations and mean field analysis for three connectivity values, ${C_{\mathrm{E}}}=5\text{,}000,\,8\text{,}000,\,11\text{,}000$. The upper plot shows the mean overall rate *ν*, and the rate of spikes belonging to waves, *ν*_W_, versus pool size *n*_E_. Although each data point represents only a single network instance, the smoothness of the behavior versus pool size indicates that fluctuations due to different network realizations are small. The lower plot shows the predicted equilibrium number of waves *h*_eq_ versus *n*_E_, as well as the mean and maximum numbers of waves obtained in the simulations. We note the good agreement between the simulations and mean field analysis. The most noticeable quantitative discrepancy is that the analysis overestimates the mean spiking rate by about 10–20 %. The reasons for this discrepancy will be discussed in Section 4.

### Measures of embedding capacity

We have defined embedding capacity relative to a given level of activity: a specified firing rate or number of waves (Section 2.5).

**Fig. 5:**
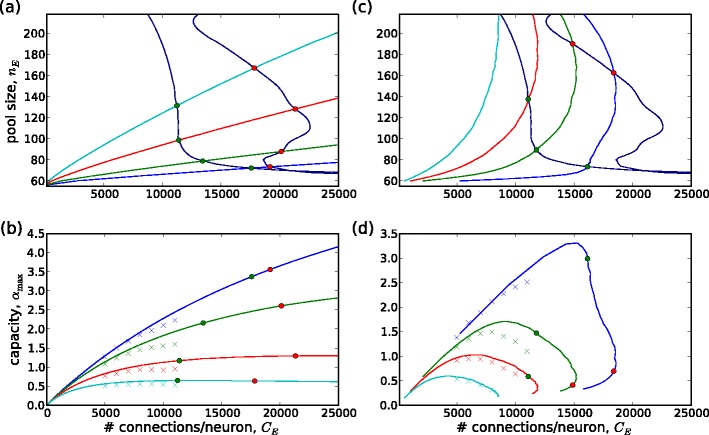
Measures of embedding capacity: (**a**) curves in (*n*_E_,*C*_E_)-space for four equilibrium firing rates (*ν*_eq_ =1,2,5,10 Hz) and for two upper limits (*C*_E,max1_(*n*_E_) for global rate stability and *C*_E,max2_(*n*_E_) for *ν*_W_/*ν* > 0.5) and the points of intersections (with *C*_E,max1_(*n*_E_) *red* ‘o’s, with *C*_E,max2_(*n*_E_) *green* ‘o’s); (**b**) corresponding embedding capacity *α*_max_(*C*_E_) curves and limits, simulation results (‘x’); (**c**,**d**) as for (**a**,**b**) but for $h_{\rm {eq}}/{N_{\mathrm{E}}}=(2,5,10,20)/80\text{,}000$

First, we give the embedding capacity based on equilibration at a particular firing rate. We consider firing rates typical of background activity in the cortex (*ν*_eq_ = 1, 2, 5, 10 Hz). Figure 5(a) shows for each *ν*_eq_ in (*n*_E_,*C*_E_)-space the curves *λ*_E,max_(*n*_E_)/*C*_E_ = *ν*_eq_. The corresponding embedding capacities *α*_max_(*C*_E_) versus *C*_E_ are shown in Fig. 5(b), along with those obtained from the simulations. The moderate quantitative discrepancy between the two is due to the discrepancy between between the predicted and observed mean firing rates seen in Fig. 4(d).

For each firing rate, the embedding capacity rises steeply with connectivity and then levels out, apparently suggesting that embedding capacity can be maximized by increasing connectivity to this level. However, two constraints on connectivity need to be respected. (Section 2.5). The first constraint limits the connectivity to the level at which the fixed point of low stochastic spiking rate becomes unstable:

18 $$ {C_{\mathrm{E}}}={C_{\mathrm{E,max1}}}({n_{\mathrm{E}}})=1/\left.\mathrm{(d}\hat{f_{\mathrm{S}}}/\mathrm{d}{\lambda_{\mathrm{E}}})\right|_{{\lambda_{\mathrm{E}}}={\lambda_{\mathrm{E,max}}}({n_{\mathrm{E}}})}. $$

The second constraint, that at least 50 % of the spiking should belong to wave activity, leads to a smaller upper connectivity limit given by *C*_E_ = *C*_E,max2_(*n*_E_) = *λ*_E,max_(*n*_E_)/2*f*_S_(*λ*_E,max_(*n*_E_)). These two upper bounds are shown in Fig. 5(a). Where they intersect the curves *λ*_E,max_(*n*_E_)/*C*_E_ = *ν*_eq_ defines the connectivity limits for each *ν*_eq_. These limits are marked by symbols on the corresponding embedding capacity curves in Fig. 5(b). Thus, taking into account the global rate dynamics shows that it is not always possible to increase the connectivity to the point where the capacity levels out. For *ν*_eq_ =1, 2, 5 Hz, maximum embedding capacity is, in fact, limited to a smaller value by the lower of the two connectivity limits, *C*_E,max2_.

Second, we consider embedding capacity based on equilibration at a particular number of waves (relative to network size). The choice of the number of waves is arbitrary. We consider $h_{\mathrm{eq}}/{N_{\mathrm{E}}}=(2,5,10,20)/80\text{,}000$ (corresponding to *h*_eq_ = 2,5,10,20 for the case ${C_{\mathrm{E}}}=8\text{,}000$). In analogy to Fig. 5(a), the curves in (*n*_E_,*C*_E_)-space of *h*_eq_/*N*_E_ along with the two upper limits on connectivity are given in Fig. 5(c). Embedding capacity versus connectivity is given in Fig. 5(d), along with the results obtained by network simulations.

The definitions of embedding capacity allow us to study how it can be optimized with respect to other parameters. In the present work, we use the definition based on the equilibrium firing rate to assess the role of inhibitory conductance.

### Inhibitory conductance governs trade-off between wave propagation stability and stochastic spiking stability

**Fig. 6:**
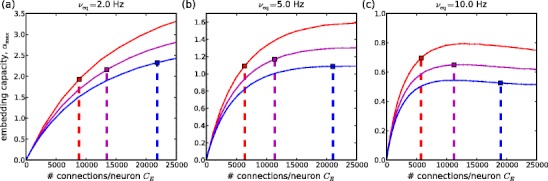
Dependence of embedding capacity on inhibitory conductance. Embedding capacity curves *α*_max_(*C*_E_) for 3 equilibrium firing rates *ν*_eq_ = 2, 5, 10 Hz (panels **a**–**c**); for *g*_I_ = 0.1 (*red*), 0.11, (*magenta*), 0.12 (*blue*). *Vertical dashed lines* show the corresponding limits on connectivity for 50 % of spikes to belong to synfire waves (*C*_E,max2_(*n*_E_))

The justification for our initial choice of the inhibitory conductance value was that it leads to a small amount of stochastic spiking, relative to wave-spiking. From the theory of balanced random networks (Brunel 2000; Meffin et al. 2004) an externally driven network must be in the inhibition-dominated regime in order to produce the low-rate, irregular firing rates typical of background activity. This regime is known as the fluctuation-driven regime, because spiking is due to fluctuations around a mean membrane potential positioned below the firing threshold. We want to consider how sensitive the tuning of this mean membrane potential is for the performance of our model. Figure 6 shows predictions of the mean field analysis for embedding capacities defined using three different choices of equilibrium firing rate: *ν*_eq_ = 2, 5, 10 Hz. For each, three choices of inhibitory synaptic conductance are examined (*g*_I_ = 0.1, 0.11, 0.12). These values are, respectively, the one used for the network simulations and a value on either side of it. All three values are well within the inhibition dominated regime.

The embedding capacity curves are higher when the inhibitory synaptic conductance is smaller. This is because a higher membrane potential distribution allows stable propagation at smaller pool sizes. This is consistent with Fig. 3(d), which shows that mininimum pool size for stable propagation decreases as inhibitory conductance increases. On the other hand, as seen in Fig. 3(e), the stochastic spiking response is stronger with reduced inhibitory conductance. Hence the upper bound on connectivity for 50 % of spikes to belong to synfire waves (*ν*_W_/*ν* = 0.5) is lowered. The consequence is that there is a trade-off between the stability of wave propagation and the control of stochastic spiking. For *ν*_eq_ = 5 Hz, Fig. 6(b) indicates the existence of an optimal inhibitory synaptic conductance close to 0.11, with maximum capacity occurring at a connectivity of about 11,000 per neuron. For a lower (higher) choice of equilibrium firing rate, the optimal inhibitory conductance is reduced (increased) and the optimal connectivity is increased (reduced), as indicated by Fig. 6(a) and (c).

### Comparison of conductance-based and current-based models

To explain the high embedding capacity of the present model compared to a current-based model such as that of Aviel et al. (2005), it is desirable to examine the behavior of the two models across suitable slices of parameter space and relate the underlying neuronal response properties of the two models to the global network measure of embedding capacity. We consider two quite different ways of making this comparison: firstly, one that explores conventional parameter choices for the current-based model; and secondly, one that uses choices specifically designed to fit the current-based model behavior to that of the conductance-based model.

In the current-based model, Eq. (2) for the conductance-based subthreshold membrane potential dynamics is replaced by:

19 $$ \frac{dV}{dt}\!=\!\frac{{V_{\mathrm{P}}}-V}{{\tau_{\mathrm{P}}}}\!+\! \sum\limits_{j,k}{a_{\mathrm{E}}}\delta\left(t\!-\!t_{j}^{k}\right)\!+\!\sum\limits_{j,k}{a_{\mathrm{I}}} \delta\left(t\!-\!t_{j}^{k}\right)\label{eq:sub-thresh-mem-pot-curr-based} $$

where *a*_E_, *a*_I_ are the amplitudes of the synaptic input responses, replacing the voltage-dependent response amplitudes, *g*_E_(*V*_E_ − *V*), *g*_I_(*V*_I_ − *V*) respectively.

To facilitate the comparison, we use a simple, approximate method of analysis which, unlike the comprehensive mean field analysis presented in Section 2.4, does not require simulations of single chains and neurons to obtain solutions. The simple analysis offers insights into the mechanisms but it comes with an important caveat: unlike the comprehensive analysis, it neglects the effects of the 0.5 ms spread in intra-link delays that is present in the model used for the chain embedding simulations, as specified in Section 2. Comparison of the two analyses—simple and comprehensive—therefore gives an indication of the impact of intra-link delay variability. The simple method uses the well-known Siegert formula, an analytic expression for the stochastic spiking rate as a function of background input, based on a diffusion approximation for the stochastic membrane potential, originally developed for current-based synapses (Ricciardi 1977; Brunel 2000) and later adapted for conductance-based synapses (Burkitt et al. 2003). In both cases it takes the form

20 $$ f_{\mathrm{S}}({\lambda_{\mathrm{E}}},{\lambda_{\mathrm{I}}})=\left[\tau_{\mathrm{ref}}+\tau\int_{\frac{{V_{\mathrm{R}}}-\mu}{\sqrt{2}\sigma}}^{\frac{{V_{\mathrm{\Theta}}}-\mu}{\sqrt{2}\sigma}}\sqrt{\pi}e^{z^{2}}\mathrm{(1+erf}(z))dz\right]^{-1} $$

where *μ* and *σ* are the mean and standard deviation of the membrane potential distribution without a spiking threshold (approximating that with the threshold) and *τ* is the *effective* membrane time constant. The dependence of *μ*, *σ*, and *τ* on the background input rates (*λ*_E_, *λ*_I_) and the neuronal and synaptic parameters is given in Burkitt (2006) for both conductance and current based models. For the current-based model *τ* = *τ*_P_, while for the conductance-based model *τ* is reduced substantially in the high-conductance state, which leads to smaller *σ* values than a current-based model with the same *τ*_P_. This is a critical feature of the conductance-based model.

In the first method of comparison, the neuronal parameters of the current-based model (*τ*_P_, *V*_P_, *V*_R_, *V*_Θ_ and *τ*_ref_) are set to the same values as used in the conductance-based model (Table 1, Section 2.2). In the second method, however, we allow the current-based model to take different values for *τ*_P_ and *V*_P_, in order to fit the membrane potential distribution and the effective membrane time constant of the current-based model to those of the conductance-based model.

For each particular choice of the neuronal and synaptic parameters of either model, we determine the chain embedding capacity of the network. Following Section 2.5, we assume the system is operating at a given mean firing rate, which we take to be *ν* = 5 Hz, and require that less than 50 % of the spikes are stochastic. As the connectivity per neuron, *C*_E_ (with *C*_I_ = *γC*_E_), is varied, we determine (a) the equilibrium stochastic spiking rate, and its stability; (b) the minimum pool size *n*_E,min_ for stable propagation at this firing rate and connectivity; and (c) the capacity (${\alpha_{\mathrm{max}}}={C_{\mathrm{E}}}/n_{\mathrm{E,min}}^{2}$). To simplify the analysis here, we neglect the background-input dependence of the rate of spikes belonging to synfire waves (Eq. (7)). This allows a simpler self-consistency equation for the system: we can specify the spike rate due to waves (*ν*_W_) independently of the amount of stochastic spiking (by setting the number of waves, *h*, appropriately) and solve for a self-consistent stochastic spiking rate *ν*_S_ such that ${\nu_{\mathrm{S}}}=f\hat{S}({C_{\mathrm{E}}}({\nu_{\mathrm{W}}}+{\nu_{\mathrm{S}}}))$ where ${\nu_{\mathrm{S}}}={\hat{f}_{\mathrm{S}}}({\lambda_{\mathrm{E}}})\equiv{f_{\mathrm{S}}}({\lambda_{\mathrm{E}}},\gamma{\lambda_{\mathrm{I}}})$. Since we require *ν*_W_ + *ν*_S_ = *ν*, the solution is obtained for any connectivity level just by setting ${\nu_{\mathrm{S}}}=\hat{f}_{\mathrm{S}}({C_{\mathrm{E}}}\nu)$ and *ν*_W_ = *ν* − *ν*_S_, provided *ν*_W_ is non-negative. This is guaranteed by our requirement that *ν*_S_ < 0.5*ν*. As before, stability of this solution is assessed using Eq. (11).

The minimum pool size for stable wave propagation, *n*_E,min_ is estimated by considering the Gaussian approximation to the membrane potential, and requiring that *n*_E,min_ synchronous inputs of amplitude *a*_E_ (current model) or *g*_E_(*V*_avg_ − *V*) (conductance model) will put 95 % of the membrane potential distribution above threshold, so that the probability of a spike response to the input is 0.95. This leads to the condition *n*_E,min_ *a*_E_ = *V*_Θ_ − (*μ* − 1.645*σ*). Importantly, however, this condition is only reasonable on the assumption of a zero intra-link delay spread. In the model specified in Section 2 there is an intra-link delay spread of 0.5 ms. The resulting dispersion of the synfire wave inputs introduces a window of integration during which background input can affect the probability that a spike is produced, so an estimate of minimum pool size based only on the membrane potential distribution prior to the arrival of synfire wave input will only be a good approximation if *τ*, the time scale at which background input acts on the membrane potential, is large in comparison to the spread of intra-link delays.

To identify the best capacity for each specific choice of neuronal and synaptic parameters, we obtain the behavior as a function of connectivity up to a maximum connectivity, *C*_E,max_, the point at which either (a) the fixed point solution for *ν*_S_ becomes unstable, (b) the condition *ν*_S_ < 0.5*ν* = 2.5 Hz breaks down, or (c) *α*_max_ ceases to increase.

We examine the behavior of the conductance based model for one choice of excitatory conductance (*g*_E_ = 0.005 as before) while the inhibitory conductance *g*_I_ is varied over the interval [0.02,0.1]. For the *first* method of comparison, a corresponding family of current-based models is formed by setting *a*_E_ to match *g*_E_ using the relation *a*_E_ = *g*_E_(*V*_avg_ − *V*) where *V*_avg_ = (*V*_R_ + *V*_Θ_)/2. Hence *a*_E_ = 0.312 mV. Choosing *a*_E_ as a function of *g*_E_ is appropriate, since the excitatory response in the conductance-based model does not vary much for $V\in\left[{V_{\mathrm{R}}},{V_{\mathrm{\Theta}}}\right]$. However, this is not true for the inhibitory response, so a one-to-one match between *a*_I_ and *g*_I_ is not well founded. Instead we simply vary the inhibitory conductance over the interval *a*_I_ ∈ [0.25, 1.5] mV and compare the two models over their respective ranges of *a*_I_ and *g*_I_, these ranges being sufficiently representive of the behavior of the two models as inhibition is varied.

**Fig. 7:**
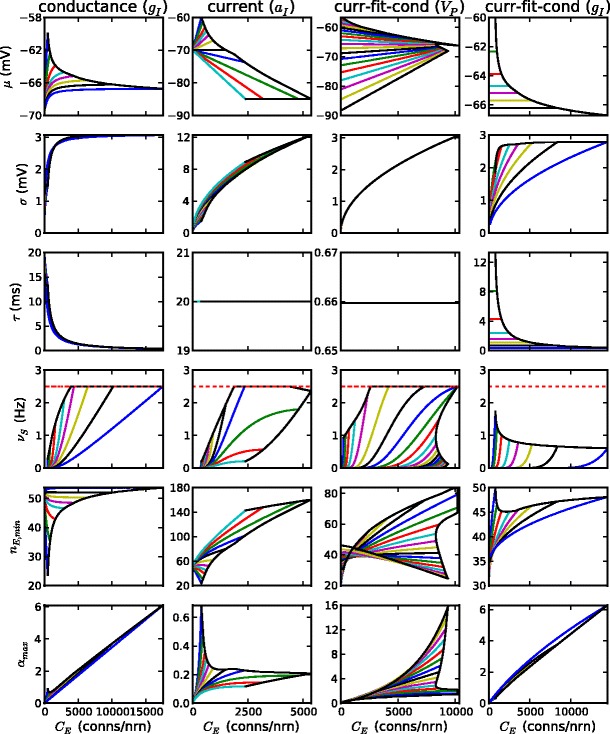
The comparison of conductance-based and current-based models: (*first column*) conductance-based models with *g*_E_ = 0.005 and *g*_I_ ∈ [0.02,0.1]; (*second column*) current-based models with *a*_E_ = *g*_E_(*V*_E_ − *V*_avg_) and *a*_I_ ∈ [0.25, 1.5] mV; (*third column*) current-based models fitted to a conductance-based model with *g*_E_ = 0.005 and *g*_I_ = 0.095, for *V*_P_ ∈ [ − 88.4, − 56.6] mV; (*fourth column*) current-based models fitted to each of the conductance models in the first column using *V*_P_ = *μ*. In each column, the remaining neuronal parameters are as given in Table 1. For selected values of the varied parameter (*g*_I_, *a*_I_, *V*_P_ and *g*_I_, for columns 1–4 respectively) the performance parameters *μ*, *σ*, *ν*_S_, *n*_E,min_ and *α*_max_ are plotted against connectivity *C*_E_ up to *C*_E_ = *C*_E,max_, the point that gives the highest embedding capacity subject to the constraints on stochastic spiking rate and stability (*colored curves*). The values of the varied parameter are: (*first* and *fourth columns*) *g*_I_ =0.02, 0.05, 0.07, 0.08, 0.085, 0.09, 0.095, 0.10; (*second column*) *a*_I_ =0.25, 0.5, 0.75, 0.875, 1., 1.125, 1.25, 1.3125, 1.375, 1.4375, 1.5 mV; and (*third column*) 21 values of *V*_P_ between − 88.4 mV and − 56.6 mV. In each panel the black curve is the behavior at *C*_E_ = *C*_E,max_ for the entire range of the varied parameter

The results of the first method of comparison are shown in the first and second columns of Fig. 7, for the conductance and current-based models respectively. At low inhibitory strengths the behavior of the two models is similar. In this region the mean membrane potential is poised not far from threshold, allowing small minimum pool sizes and therefore moderately high embedding capacities. However, this region is not realistic in that it only permits a low level of connectivity, because inhibitory feedback strength is insufficient to control recurrent excitation. Moreover, this region is outside the balanced, inhibition-dominated regime of AI spiking activity that (for this class of models) best represents activity in the cortex. When we come to higher inhibitory strengths the two models diverge dramatically. In the current-based model, as connectivity increases the mean membrane potential drops steadily and the standard deviation widens, leading to unfavourably large minimum pool sizes and low embedding capacities. In the conductance-based model the mean membrane potential and standard deviation approach asymptotic values and hence so does the minimum pool size, leading to an embedding capacity that increases linearly with connectivity (since $\alpha={C_{\mathrm{E}}}/n_{\mathrm{E}}^{2}$). As inhibition increases, embedding capacity optimized with respect to connectivity decreases in the current-based model and increases linearly in the conductance-based model, so that cortical-scale connectivity is only obtainable in the latter.

In Fig. 7 the horizontal axis can also be intepreted to represent the background input rate, via the conversion *λ*_E_ = *C*_E_ *ν*_eq_. In particular, we can see the behaviour of *n*_E,min_ versus *λ*_E_. For both conductance and current-based models, at low inhibitory strengths *n*_E,min_ decreases with background rate, implying that wave activity is not regulated by background input. At higher inhibitory strengths *n*_E,min_ increases with background rate for the current-based model, and thus wave regulation is restored. However, for the conductance-based model the membrane potential distribution approaches a limit and virtually ceases to change as background input increases, and hence *n*_E,min_ reaches an asymptotic value at *C*_E_ values well below *C*_E,max_, implying that wave regulation is ineffective. This result is in contrast to our findings for a conductance model with a 0.5 ms spread in intra-link delays: in the network simulations there *is* effective regulation of the number of waves (Fig. 2) and the comprehensive mean field analysis (Section 3.2, Fig. 3) shows that an increase in background input reduces the probability of a spike in response to synfire wave input and increases the minimum pool size. These effects, being absent in a model with zero intra-link delay spread, must be due to the effect of background input during the window of integration of synfire wave input that exists when there is a 0.5 ms intra-link delay spread. By increasing the minimum pool size, the intra-link delay spread also reduces the embedding capacity, as can be seen by comparing embedding capacity curves in Figs. 5(b) and 6 (0.5 ms delay spread) with those in the first column of Fig. 7 (zero delay spread). With zero delay spread the capacity increases linearly with connectivity because the minimum pool size is asymptotically constant. When the delay spread is introduced, the capacity curves become concave down in shape, due to a minimum pool size that increases almost linearly (with an offset) as connectivity increases (Fig. 3(d)). The large effect of intra-link delay spread in the conductance model is not surprising, since *τ* drops rapidly with *C*_E_ and is already within an order of magnitude of the intra-link delay spread at ${C_{\mathrm{E}}}=1\text{,}000$. However, the findings for the current-based model (Fig. 7, second column) should be only slightly changed by a 0.5 ms intra-link delay spread, since *τ*_P_ is 40 times larger than that.

Turning now to the *second* method of comparison, here we choose parameters for the current based model that will fit the effective membrane time constant and membrane potential distribution to that of a given conductance model at a given mean firing rate *ν* and recurrent connectivity *C*_E,fit_. We set *C*_E,fit_ to be the *C*_E,max_ of the conductance-based model, since that is where, overall, its best capacities are found. *ν* and *C*_E,fit_ determine *λ*_E_ and *λ*_I_ and hence *μ*, *σ* and *τ*. For the current-based model, we set *τ*_P_ = *τ* and constrain *a*_E_, *a*_I_, and *V*_P_ to yield the same *μ* and *σ* as the conductance model. Since the constraints leave one degree of freedom, they leave *a*_E_ and *a*_I_ as functions of *V*_P_. Increasing *V*_P_ decreases *a*_E_/|*a*_I_| and vice versa, leading to a trade-off between rate stability and wave regulation on the one hand, and capacity on the other. The third column of Fig. 7 shows for a range of *V*_P_ values the performance of the current-based model fitted to one particular conductance-based model (*g*_I_ = 0.095). For low *V*_P_, the mean membrane potential increases with connectivity and the minimum pool size decreases. Thus, the embedding capacity increases but there is no regulation of wave activity. For high *V*_P_, the opposite is the case: there is regulation but capacity is low. There is a narrow range of choices for *V*_P_ where there is both high capacity *and* regulation of the number of waves. One such value is *V*_P_ = *μ*. This is understandable because *V*_P_ = *μ* implies *a*_E_ *λ*_E_ + *a*_I_ *λ*_I_ = 0. Hence *μ* remains unchanged as the number of waves and the mean rate increase. Thus the balanced state is preserved (as in the conductance model), while *σ* increases (unlike the conductance model) resulting in a gradual increase in the minimum pool size (which must shift 95 % of the membrane potential distribution above the firing threshold). Therefore it is charitable to choose *V*_P_ = *μ* to fit a particular current model to each conductance model. The fourth column of Fig. 7 shows the performance of a current-based model fitted in this way to each of the conductance-based models shown in the first column, as *g*_I_ is varied. We observe that the current-based model behaves similarly to the conductance model with respect to the membrane potential parameters (*μ*, *σ*) and the capacity. However, unlike the conductance model, the limit *C*_E,max_ (which determines the ’envelope’ curve) is not due to the 0.5*ν* limit on the stochastic spiking rate (*C*_E,max2_), but to the limit at which stochastic spiking becomes unstable (*C*_E,max1_). Thus the drop in performance is not graceful as connectivity increases (with the amount of wave activity decreasing due to regulation), but catastrophic. This could only be remedied by choosing a higher *V*_P_, as the third column shows, at the expense of reducing the capacity.

A difference between the conductance and current-based models, the latter with the values of *μ*, *σ* and *τ* fitted to the former, lies in the origin of wave regulation. In both models, zero intra-link delay spread behavior is a poor match to that with 0.5 ms intra-link delay spread, because the effective membrane time constant is small. A strong drift rate − (*V* − *μ*)/*τ* impedes the rise to threshold during the integration of synfire wave input, leading to a larger minimum pool size with the 0.5 ms intra-link delay spread. In the conductance-based model, the small *τ* is dynamic, and due to background input: 1/*τ* = 1/*τ*_P_ + *λ*_E_(*g*_E_ + *γg*_I_). In the fitted current models, however, *τ* is small but static: *τ* = *τ*_P_. Wave regulation requires that the minimum pool size increase with background input. In the conductance-based model this happens because *τ* decreases even more, while *μ* and *σ* remain almost unchanged. In the current-based model, *τ* cannot change, so regulation must be due to the effects of background input on *μ* and *σ*. The conditions for regulation are essentially the same for 0.5 ms intra-link delay spread as for zero intra-link delay spread. We need *μ* to change only slightly, together with an increase in *σ*, to ensure that *n*_E,min_ increases. The conclusion that there is a narrow range of parameters for the current-based model that allow both capacity and wave regulation, including values *V*_P_ ≈ *μ* and *a*_E_ *C*_E_ + *a*_I_ *C*_I_ ≈ 0, is not qualitatively affected by the introduction of 0.5 ms intra-link delay spread.

In conclusion, the comparison of conductance and current-based models has shown that the former outperforms the *conventional* current-based model because the latter, with a membrane time constant in the order of tens of milliseconds, overestimates the magnitude of membrane potential fluctuations in the high conductance state, which leads to a very poor tradeoff between controlling stochastic spiking and keeping small the minimum pool size for wave propagation. A current-based model with the effective membrane time constant and membrane potential distribution matching those of the conductance model shows similar performance to the latter, both in capacity and regulation of waves. The simple analysis of this section assumed an intra-link delay spread of zero. If the intra-link delay spread is non-zero, the capacity of both the conductance model and the current-based model with the effective time constant fitted to that of the conductance model will be substantially reduced, but they still well-outperform that of the conventional current-based model.

## Discussion

We presented a cortical network model that allows for the embedding of large numbers of pools linked to form synfire chains. Key features of this model are (i) use of conductance-based synapses, (ii) incorporation of inhibitory pools of neurons into the chains so that synfire waves conserve the balance of excitatory and inhibitory activity, and (iii) variable intra-link transmission delays. The number of pools is at the combinatorial capacity for a given connectivity per neuron: all excitatory connections are used to constitute pool-to-pool links.

We gave a mean-field analysis that determines steady state properties (mean firing rate and components due to stochastic spiking and spikes belonging to synfire waves) as a function of model parameters. Performance is assessed in terms of number of pools and number of co-active waves. There are two dynamical constraints on performance: stability of synfire wave propagation and stability of the low rate AI state of overall network activity. The analysis determines the boundaries for satisfying these two constraints. The constraint of synfire wave stability amounts to a minimum pool size for a given level of background input. For the constraint of AI stability, we provide a “naïve” estimate based on stability of the AI state of the corresponding balanced random network driven by balanced input. This is a necessary condition, but it may not always be sufficient, as in the case for the model studied by Aviel et al. (2005) for instance. Hence we use numerical simulations to confirm AI stability.

The mean field analysis (Section 3.2) shows (for three choices of inhibitory strength) that the minimum pool size increases with background input; equivalently, there is an upper limit on background input for wave propagation at a given pool size. Since wave activity drives background input, regulation of wave activity emerges as a natural outcome of the model: there is an upper bound on the number of propagating waves. In our simulations we drive the system to equilibrium at this upper bound since the pool size is then at the minimum (and the embedding level is at the maximum) for the resulting mean firing rate at the given connectivity per neuron. We verify that over a wide range of pool sizes and connectivity levels the AI state remains stable at this equilibrium and the mean field analysis provides a good quantitative description of the equilibrium behavior. We find a trade-off between embedding capacity on the one hand, and both equilibrium firing rate and equilibrium number of co-active waves on the other hand. Therefore, we define embedding capacity with respect to either the equilibrium firing rate or the equilibrium number of waves. For cortical scale connectivity in the range of 5,000–11,000 excitatory synapses per neuron, we obtain embedding capacities of around 0.5–2.0 for firing rates of 1–10 Hz and for 2–10 waves per 80,000 neurons respectively.

These capacity results are substantially higher than those found in previous models of chain embedding using spiking neurons and current-based synapses (Aviel et al. 2005; Schrader et al. 2008). The higher embedding capacity of the present model is a direct result of two outcomes: a small minimum pool size for a given connectivity, and a high connectivity attainable without a ground state instability. The improvement in both of these outcomes can, according to the analysis made in Section 3.7, be attributed to the use of conductance-based synapses. However, the predicted increase in the attainable connectivity is based on the stability of the mean spike rate of a balanced random network. The naïve prediction will only be valid if the chain-embedding network is no more susceptible to a breakdown of the AI state of spiking activity than the corresponding random network. Our simulation results are consistent with this supposition, but they do not go all the way to limit. Exactly why the present network avoids instabilities in the AI state that occur in previous studies, such as the synfire explosion (Mehring et al. 2003; Aviel et al. 2005), is not clear. The three key ingredients of the model (conductance-based synapses, variability in delays, and balanced pools) are all implicated in this improvement of AI stability but we have not teased apart their relative contributions. Network size is also important for high capacity. Figures 5 and 6 clearly indicate that high capacity is only attained when the connectivity per neuron reaches several thousand.^8^

The structure of inhibition in the model serves to keep the network in a balanced state, where the ratio of the excitatory and inhibitory input rates and the mean membrane potential remain approximately constant as the number of waves (and the overall firing rate) increase due to external stimulation, by ensuring that excitatory and inhibitory mean firing rates are co-modulated, and indeed remain equal. This is achieved by (a) E–I balanced pools; and (b) the same mean recurrent connectivity to both E and I neurons. (a) and (b) respectively ensure that the mean rate of spikes belonging to synfire waves and the stochastic spiking rate are the same for both E and I neurons. The strength of inhibition affects capacity in two ways. On the one hand, increasing inhibition reduces capacity by increasing the required pool size at a given connectivity, but on the other hand, it allows for more recurrent connectivity by reducing stochastic spiking in response to noise. Overall there is a trade-off between these two effects which determines an optimal inhibition, and this optimum will decrease if the desired equilibrium firing rate is increased.

Our model has structural symmetries that cause the firing rates of excitatory and inhibitory neurons to be the same, for simplicity. This will not be the case if *C*_E_, *C*_I_, *g*_E_ or *g*_I_ differ for the synaptic inputs of the two neuron types, or if the condition for balanced pools, *n*_E_/*N*_E_ = *n*_I_/*N*_I_, is not satisfied. This will not change the qualitative properties of the model if the ratio of the excitatory and inhibitory spiking rates remains approximately invariant. However, it would be interesting to break the symmetry in such a way that the stochastic spike rate increases more rapidly for inhibitory neurons than for excitatory ones, as this might allow stronger regulation of the number of waves at the same upper bound.

A simplified mean field analysis was used to make an efficient comparison of the conductance-based and current-based models for varying inhibitory strength and connectivity (Section 3.7). Two methods for specifying the current-based model were examined. For the first method, in which conventional parameters for the current-based model are used, the two models differ strikingly in behavior: optimal embedding capacity decreases with respect to inhibition for the current-based model but increases in an asymptotically linear manner for the conductance-based model. The difference stems from the behaviour of the membrane potential distribution. First, at high connectivities in the conductance-based model the neuron is in the high-conductance state in which the effective membrane time constant is substantially reduced (Destexhe and Paré 1999; Meffin et al. 2004; Richardson 2004), leading to a narrower membrane potential distribution than in the current-based model. Second, the inhibitory reversal potential prevents the mean membrane potential from becoming too low. Combined, these effects allow a neuron with conductance-based synapses to maintain both a high response to coincident input and a low response to background input as connectivity increases. This in turn allows cortical scale connectivity to be attained without substantial increase in pool size. For the second method, the current-based model is given a very small membrane time constant to match the effective membrane time constant of the conductance-based model, and other neuronal and synaptic parameters are constrained to fit the membrane potential distribution to that of the conductance-based model. Within these constraints there is a trade-off between capacity and wave regulation, and there exists a small range of parameters which achieve both wave regulation and capacities similar to those of the conductance model.

The model we have presented here uses delta-function synaptic responses and includes variability in the excitatory transmission delays within each synfire link in the form of a 0.5 ms intra-link delay spread. The comprehensive mean field analysis of Sections 2.4 and 2.5 takes this into account. However, the simplified analysis of Section 3.7 is only valid for a model with instantaneous synaptic responses and zero spread of intra-link transmission delays. As discussed in Section 2.2, this is not realistic as it leads to the omission of an integration window during which background input can prevent the spike response to synchronous input. Such a window will be present in a model with a spread of intra-link delays. The intra-link delay spread plays a critical role in determining the embedding capacity. We have shown that intra-link delay variability reduces robustness of wave propagation and hence capacity, but that such variability is necessary in a model with instantaneous synaptic responses in order that waves are not so robust that they cannot be regulated. Section 3.7 shows that regulation is missing in the conductance-based model with zero delay spread. Thus the delay spread governs a trade-off between capacity and regulation. It is also responsible for the existence of an optimal level of inhibition (Section 3.6); without a delay spread, embedding capacity simply continues to increase with increasing inhibition, along with connectivity and network size (Fig. 7, first column). Even without intra-link delay spread, a window of integration of synfire input will be present if synaptic responses are non-instantaneous (ie. there are post-synaptic rise times). Post-synaptic rise times should have the same effects as intra-link delay spread: regulation of wave activity in exchange for reduced capacity, and the existence of an optimal level of inhibition. Being more realistic than instantaneous synaptic responses, they should be included in future studies.

Apart from the role of the intra-link excitatory delay variability, how else do our results depend on the excitatory and inhibitory delay distributions used? The mean excitatory delay excitatory is important as the main component of the pool-to-pool propagation time, and thereby strongly affects how many waves can simultaneously propagate. If the mean delay is increased, the rate of spiking due to a single wave decreases and thus the number of waves will increase while the mean-field solution for the rates (*ν*_W_, *ν*_S_, etc.) remains invariant. The model would not support as many waves if the mean delay was shorter than the 2.5 ms value used, which we argued was realistic for local cortical connections. The inhibitory delays don’t play any direct role in synfire propagation but differences in E and I delay distributions will affect the nature of the steady state of the balanced random network (Brunel 2000). The asynchronous-irregular state of stochastic spiking is stable for the delay distributions we used, but with different distributions it could give way to synchronous-irregular (SI) oscillations. An interesting question for further study is the extent to which synfire propagation is compatible with SI oscillations.

Some simplifications made in the mean field analysis of Section 2.4 may explain the modest deviations from the simulation results. The analysis treats the stochastic spiking response to background input as that of a neuron receiving *solely* background input. Likewise the effects of background input on wave propagation are treated by considering a chain in which, apart from the inputs due to connections within the chain itself, the neurons received solely background input. Both treatments do not take into account that synchronous input packets also occur due to the fact that each neuron belongs to many pools in the embedding network. Synchronous inputs lead to additional spikes that cause the membrane potential of each neuron to spend a greater proportion of time in the post-spike recovery-from-reset phase, during which both the propensity to emit stochastic spikes and to respond to synchronous input from a wave propagating down the embedded chain are reduced. Taking these additional spikes into account would lower the mean field prediction for both the rate of stochastic spiking (*f*_S_) and wave propagation reliability (*P*_S_). Nevertheless, the treatments are still useful approximations: because the effective membrane time-constant of neurons in the high-conductance state is short (a few milliseconds or less) the recovery from reset to equilibrium is quick, minimizing the effect of the additional spikes. The analysis also overestimates the excitatory background input rate by including as background those inputs which are in fact synchronous: the approximation in Eq. (4). This simplification, like the previous one, leads to an overestimate of the stochastic spiking rate (*f*_S_) and wave propagation reliability (*P*_S_). Nevertheless, the approximation is reasonable, because the relative overestimate *n*_E_/(*C*_E_(1 + *ν*_S_/*ν*_W_)) is small: ~0.4–4.0 % for the cases shown in Fig. 4(d). These simplifications may explain the quantitative discrepancies between theory and simulations observed in Fig. 4(d). We see, firstly, that total spike rate is lower than predicted. This is consistent with a less-than-predicted wave propagation reliability, implying a lower *λ*_E,max_(*n*_E_) and equilibration at a lower firing rate. Secondly, the fraction of spikes which are stochastic (*ν*_S_/*ν*) is less than predicted, indicating that at a given firing rate the background input is producing less stochastic spiking. Note that the overestimates of *ν* and *ν*_S_ approximately cancel out, in so far as the predicted rate of wave-spiking (*ν*_W_ = *ν* − *ν*_S_), yields a good quantitative agreement with the model simulations. The predicted number of waves is also in good agreement with the simulations, indicating that the relationship between *ν*_W_ and *h*/*N*_E_, determined by *p*_f_(*λ*_E_)/*T*(*λ*_E_) evaluated at *λ*_E_ = *λ*_E,max_, is preserved.^9^ From the modest size of the discrepancies observed in Fig. 4(d) and the fact that their origin can explained by the simplifications made, we may conclude that the mean field theory captures the essential behavior of the model, despite the complexity of its dynamics.

The use of independent Poisson input trains for the simulations of the non-embedded chains should also be questioned. Since the inputs actually arise from sampling a common embedding network of finite size (viz. *N*_E_ = 10*C*_E_, *N*_I_ = 10*C*_I_) the neurons will have some common inputs and their background input trains will thus be correlated. Tetzlaff et al. (2004) showed that input correlations based on sampling a common reservoir of Poisson neurons greatly reduced the maximum pool size for ground state stability to a level barely above the minimum pool size for stable wave propagation. However, it was found that this effect surprisingly disappeared when the Poisson reservoir was replaced by a Brunel-type balanced recurrent network. The decorrelating effect of recurrent inhibition has recently been identified as the mechanism responsible for this (Tetzlaff et al. 2012). This may explain why the Poisson approximation for background input turns out to be quite adequate for the present model.

In this paper, because we are concerned with embedding capacity, we have focused on the situation where the number of synfire waves is driven to the maximum level by ongoing spike packet stimulation, reaching an equilibrium (Section 3.3). However, the system need not operate in this marginal regime. It supports ongoing propagation of any number of waves below the maximum. In a more structured system where there is interaction and computation among the chains, the number of waves might vary in the course of this computation, being sometimes below the limit. Furthermore, in a model in which input convergence varies over chains the upper bound on background input rate would not be global, but specfic to individual chains or portions of chains. The equilibrium rate and number of waves would then depend on the specific pattern of activation, and hence could evolve during the course of a computation.

In synfire chains the neurons are organized into discrete pools. This feature, while convenient for study and analysis, can be criticized as artificial. However, it is not essential, as synfire chains can be generalized to a class of feedforward structures known as braids (Bienenstock 1995) or polychrony (Izhikevich 2006). In these structures the neurons need not be grouped into discrete pools, and they support precisely-timed and reproducible sequences of spikes that need not be grouped into synchronous packets. For instance, a *uniform* braid generates a sequence of spikes distributed uniformly over the interval of propagation, in a steady stream. In braids, the delays on the feedforward connections are specifically related to the spike sequences so as to preserve the key property of synfire chains that, during spike sequence re-activation, each neuron receives synchronously arriving spikes from upstream neurons. In models of the emergence of feedforward structures through synaptic plasticity there is no mechanism that would enforce a pool organization, so braids may be may easier to realize biologically than chains.

The present chain-embedding model can be generalized to a braid-embedding model. To embed uniform braids, instead of assigning neurons group-wise to a sequence of pools, they can be assigned one by one to a sequence of putative spikes distributed uniformly over a time interval. Excitatory feedforward connections can then be assigned appropriate delays so that if upstream neurons fire at the assumed times, then each downstream neuron will receive near-synchronous inputs shortly before its putative spike time. The property of double-balance can be incorporated straightforwardly.

A braid has no pools, so embedding capacity based on the number of pools makes no sense for a braid. To compare the embedding level of a braid embedding to that of a chain embedding, we can define the effective pool size of a braid as that of an “equivalent chain”: one with the same input convergence and mean transmission delay, and for which a propagating wave generates the same mean spike rate. The number of pools is then the number of putative spikes divided by the effective pool size. Note that in this equivalent chain, unlike the chains in present study, the input convergence may not equal to the pool size. However, it is straightforward to generalize the embedding capacity analysis to such chains (Trengove 2006). The mean field theory given here would predict for the braid model and the equivalent chain model quantitatively similar relationships between input convergence, effective pool size, connectivity per neuron, mean firing rate and number of waves, implying similar embedding capacities too.

The present study assumes that there are biological mechanisms for forming synfire chains (or braids) but this has not been established yet. There have been model studies demonstrating the growth of feedforward structures based on spike-timing dependent plasticity (STDP) or other plasticity mechanisms, but these have operated well below the cortical scale and some include artificial constraints (Bienenstock 1991; Doursat and Bienenstock 2006b, a; Jun and Jin 2007; Fiete et al. 2010; Waddington et al. 2011). A recent study using weight-dependent STDP in a cortical-scale balanced random network architecture failed to find the growth of structure (Kunkel et al. 2011), but it is still an open issue whether a more realistic model with the right ingredients to generate structure formation will eventually be found.

An important question is whether there are experimentally accessible features of the present model that can be used to test it against neural data, and thereby infer the presence of synfire activity or challenge the model. One observable consequence of the model is that, because most spikes are due to synfire waves, the spike-triggered average (STA) of the membrane potential will show a sudden rise just before a spike. Positive evidence of this kind was found for synfire chains in the HVC nucleus of the finch (Long et al. 2010). In the cortex, one study (London et al. 2010) found such sharp rises to be vary rare, but that may be due to the anaesthetized state in which the recordings were made. A necessary control for sharp rises as evidence for synfire chains is the conductance-based balanced random network, since in that model, noise-driven spiking with a short effective membrane time constant suggest that most spikes will be due to inputs arriving synchronously by chance; therefore the STA in such a network would also show a sudden rise before the spike. Another feature of the present model is the presence of sharp peaks in the cross-correlograms of pairs of neurons when there is repeated synfire wave transmission over the sequence of pools linking the pair (data not shown). The number of pairs showing such peaks, the size of the peaks and the distribution of offsets depend on the number and duration of the waves and how they are distributed over the chains, which in turn depend on the stimulation protocol. Strong peaks have also been observed experimentally but the time offset of the peak is typically small (Funahashi and Inoue 2000; Mizuseki et al. 2009; Isomura et al. 2009). The absence in experimental data of strong peaks offset by more than a few milliseconds implies that the experiments did not observe two neurons in a frequently re-activated chain. Thus any frequently reactivated chains must include only a small proportion of the neural population being sampled.^10^

In general, the experimental detection of synfire activity is a difficult task, but methods for its detection have been developed recently (Schrader et al. 2008; Gerstein et al. 2012). These methods require multi-neuron recordings and repeated activation of chains. The number of simultaneously recorded spike trains required for reliable detection has been estimated at several hundred, assuming both the recording sites and the chains are co-localized to a volume of ∼ 10^5^ neurons. With less localization more parallel recordings are required. The methods have been tested on data generated by a variant of the present model, and clearly reveal the presence of synfire activity (Gerstein et al. 2012).

To conclude, we have considered the problem of constructing a model for embedding many entities (synfire chains) in a cortical scale network in which many of them may be activated simultaneously (by waves). With respect to the two aspects of capacity, namely number of pool-to-pool links and number of co-active waves, the present model achieves a performance level that sets a new world record for spiking neuron models. For realistic synaptic strengths, connectivity at the cortical-scale optimizes performance and is achieved while maintaining global stability of rate, without the need for a global mechanism to impose a limit on activity. The model allows this improved performance for two reasons. First, the use of conductance-based synapses compared to current-based increases the contrast between the neuronal response to synchronous input signal and the response to background noise. Second, instability of the global state of asynchronous irregular spiking is avoided by using balanced synfire waves and heterogeneity of transmission delays.

## Footnotes

1 Another, current-based, network model exhibits simultaneous propagation of two waves in a network of 50,000 neurons (Schrader et al. 2008). In this model the combinatorial capacity is reached, but the connectivity is well below the cortical scale and the number of pools is only 0.02*N*. This number is in fact at the capacity limit, above which the background activity becomes unstable (Diesmann, private communication).

2 A normalized conductance response is the time-integrated synaptic conductance response divided by the capacitance, and is dimensionless.

3 Thus the stimulus has the same effect on the target pool as would a packet of *n*_E_ spikes of standard deviation 0.1 ms occurring in the previous pool.

4 In Section 3.3 we will describe an equilibrium *with* ongoing external stimulation of new waves, in which the number of waves is maintained at stable equilibrium level, *h*_eq_, because the rate of wave births is balanced by a corresponding rate of wave deaths.

5 The excitatory background input rate *C*_E_((1 − *n*_E_/*C*_E_)*ν*_W_ + *ν*_S_) is the expected value for a neuron. For a specific neuron or a specific time interval the rate will deviate from this. However, the standard deviation of the rate is small relative to the mean. In particular, even if the pools are visited by waves very unequally in frequency (as will be the case with the wave stimulation protocol of Section 2.3) the neurons still receive similar background input rates, because the background input is summing the input of *C*_E_ neurons, each of which contributes spikes belonging to spike packets in *C*_E_/*n*_E_ − 1 pools. Thus the coefficient of variation (across neurons) of the contribution to background input due to spikes belonging to spike packets is approximately $1/\sqrt{({C_{\mathrm{E}}}({C_{\mathrm{E}}}/{n_{\mathrm{E}}}-1))}\approx10^{-3}$ times the coefficient of variation (across pools) of the rate at which a pool is visited by waves.

6 This is an adequate condition for the stability of the stationary rate of a balanced random network driven by external balanced external input at rates *C*_E_ *ν*_W_ and *C*_I_ *ν*_W_, except where the latter exhibits an oscillatory instability arising from a Hopf bifurcation. Note, this formulation neglects the dependence of *ν*_W_ on background input.

7 For notational brevity, and since *λ*_I_ = *γλ*_E_ , the *λ*_I_-dependence of *T*_S_,*P*_S_,*p*_f_,... is left implicit in this section.

8 The connectivity needed for high capacity can be reduced by using larger synaptic strengths (Trengove 2006) but not by very much, as synaptic strengths an order of magnitude larger than used here would be biologically implausible and would cause the diffusion approximation breaks down, leading to more stochastic spiking than predicted by Eq. (20), and hence less increase in capacity.

9 The theory overestimates *p*_f_/*T*, but since *p*_f_/*T* decreases with *λ*_E_, this is compensated for by the overestimate in *λ*_E,max_.

10 An upper bound on how frequently a chain can be re-activated without producing a visible peak could be estimated by the condition that peak be less than twice the background level. Given a binsize *δt* and a mean firing rate *ν* this implies a chain activation rate less than *ν* ^2^ *δt*. For *ν* = 10 Hz and *δt* = 0.1 ms, chain activation rate must be below 0.1 Hz to escape detection. By that criterion, if the waves are uniformly distributed over the chains they will escape detection in the present model, but if a chain’s frequency of activation is an order of magnitude or more above the mean, then a pair of neurons in that chain would produce a detectable peak. (Note, for this discussion we are considering there to be many short chains (of order 100 pools in length) rather than a single long one (of order *N*_E_ pools) as was used in the simulations.)
